# Revealing a Two-Loop Transcriptional Feedback Mechanism in the Cyanobacterial Circadian Clock

**DOI:** 10.1371/journal.pcbi.1002966

**Published:** 2013-03-14

**Authors:** Stefanie Hertel, Christian Brettschneider, Ilka M. Axmann

**Affiliations:** 1 Institute for Theoretical Biology, Charité-Universitätsmedizin Berlin, Berlin, Germany; 2 Mathematical Modelling of Cellular Processes, Max Delbrück Center for Molecular Medicine, Berlin, Germany; Ecole Normale Supérieure, France

## Abstract

Molecular genetic studies in the circadian model organism *Synechococcus* have revealed that the KaiC protein, the central component of the circadian clock in cyanobacteria, is involved in activation and repression of its own gene transcription. During 24 hours, KaiC hexamers run through different phospho-states during daytime. So far, it has remained unclear which phospho-state of KaiC promotes *kaiBC* expression and which opposes transcriptional activation. We systematically analyzed various combinations of positive and negative transcriptional feedback regulation by introducing a combined TTFL/PTO model consisting of our previous post-translational oscillator that considers all four phospho-states of KaiC and a transcriptional/translational feedback loop. Only a particular two-loop feedback mechanism out of 32 we have extensively tested is able to reproduce existing experimental observations, including the effects of knockout or overexpression of *kai* genes. Here, threonine and double phosphorylated KaiC hexamers activate and unphosphorylated KaiC hexamers suppress *kaiBC* transcription. Our model simulations suggest that the peak expression ratio of the positive and the negative component of *kaiBC* expression is the main factor for how the different two-loop feedback models respond to removal or to overexpression of *kai* genes. We discuss parallels between our proposed TTFL/PTO model and two-loop feedback structures found in the mammalian clock.

## Author Summary

Many organisms possess a true circadian clock and coordinate their activities into daily cycles. Among the simplest organisms harboring such a 24 h-clock are cyanobacteria. Interactions among three proteins, KaiA, KaiB, KaiC, and cyclic KaiC phosphorylation govern the daily rhythm from gene expression to metabolism. Thus, the control of the *kaiBC* gene cluster expression is important for regulating the cyanobacterial clockwork. A picture has emerged in which different KaiC phospho-states activate and inhibit *kaiBC* expression. However, the mechanism remains to be solved. Here, we investigated the impact of each KaiC phospho-state on *kaiBC* expression by introducing a model that combines the circadian transcription/translation rhythm with the KaiABC-protein oscillator. We tested 32 combinations of positive and negative transcriptional regulation. It turns out that the *kaiBC* expression and KaiC phosphorylation dynamics in wild type and *kai* mutants can only be described by one mechanism: threonine and double phosphorylated KaiC hexamers activate *kaiBC* expression and the unphosphorylated state suppresses it. Further, we propose that the activator-to-repressor abundance ratio very likely determines the *kaiBC* expression dynamics in the simulated *kai* mutants. Our suggested clock model can be extended by further kinetic mechanisms to gain deeper insights into the various underlying processes of circadian gene regulation.

## Introduction

Photoautotrophic organisms like plants and cyanobacteria are subjected to a daily light-dark rhythm and have been demonstrated to possess a self-sustained circadian clock. The simplest circadian clock ticks in cyanobacteria. It consists of just three proteins KaiA, KaiB and KaiC composing a post-translational oscillator (PTO). This unique three-protein clock is well described for *Synechococcus elongatus* PCC 7942 (hereafter *Synechococcus*). The principal protein of the PTO is KaiC combining three intrinsic enzymatic activities, autokinase, autophosphatase and ATPase [1], [2]. ATPase and kinase/phosphatase occur in the C1 and C2 rings of the KaiC hexamer, respectively. KaiC hydrolyzes ∼15 ATP molecules daily [1]. The consensus view is that the ATPase crosstalks with the kinase/phosphatase through a structural coupling between the two rings [3]. KaiA promotes and KaiB represses phosphorylation of KaiC. The three Kai proteins form stable complexes during the subjective night [4], [5]. KaiC forms hexamers and each KaiC monomer within the hexamer possesses two main phosphorylation sites (T432 and S431) [6]. The four forms of KaiC cycle in a stepwise fashion: unphosphorylated (U-KaiC), threonine phosphorylated (T-KaiC), both residues phosphorylated (D-KaiC), and serine phosphorylated (S-KaiC) [7], [8].

In the presence of ATP, the three proteins KaiA, KaiB and KaiC are able to produce robust, temperature-compensated 24 h-cycles of KaiC phosphorylation even in a test tube. In the cell, KaiABC can drive the circadian transcriptional output without *de novo* expression of the *kai* genes [2], [9], [10]. Thus, the basic timing mechanism in cyanobacteria has been suggested to rely on post-translational processes whereas in eukaryotic circadian systems it is assumed to based upon transcriptional/translational feedback loops. However, with the discovery of a cellular clock in human red blood cells and in the alga *Ostreococcus tauri* that might keep time using the rhythms of metabolism, O'Neill and colleagues [11], [12] contribute to a re-definition or at least a refinement of biological timing mechanisms in eukaryotes that gain more and more similarities to that found in cyanobacteria.

Various modeling approaches have been applied to the KaiABC protein system to simulate the chemical network that is able to generate self-sustained oscillations, reviewed by Johnson et al. [13] and Markson and O'Shea [14]. Beside two other studies [7], [15], we could recently show with a quantitative, highly nonlinear feedback model that oscillations in the Kai system are a consequence of KaiA sequestration by serine phosphorylated KaiBC complexes [16], [17]. Robustness of oscillations against concerted changes in Kai protein levels is a result of the fact that most KaiA is inactive throughout the circadian cycle. Native mass spectrometry further revealed the existence of three KaiC binding sites for constant and phosphorylation-dependent sequestration of KaiA and allowed us to establish a detailed map of the complex formation dynamics [16].

Progress has been made as well in unraveling the molecular clock components that drive the observed global rhythms of promoter activity, although the picture is not yet complete. The consensus view is, that several factors function in the clock output pathways, including SasA, RpaA, LabA and CikA [18]–[21]. A recent study showed that an additional response regulator, RpaB, is also a key regulator of the circadian output pathway [22]. These output factors also play an important role in the regulation of *kaiBC* expression. Further factors (Pex, LdpA, CikA, NhtA, PrkE, IrcA, CdpA) have been revealed that may contribute to the clock input pathway. They modulate the functioning of the KaiABC protein clock [23]–[25]. A complementary scenario for circadian regulation of global gene expression is, that the daily fluctuation of chromosomal compaction and DNA supercoiling might influence promoter activity [26]–[28].

The regulation of *kaiBC* expression plays an important role in regulating the cyanobacterial circadian clockwork [29]. In *Synechococcus*, the three clock genes, *kaiA*, *kaiB* and *kaiC* are arranged as three adjacent genes. The *kaiB* and *kaiC* genes are expressed as a dicistronic operon, while the *kaiA* gene possesses an own promoter. The *kaiA* transcript is rhythmically abundant but not its protein [30]. In contrast, the *kaiBC* transcripts and the KaiB and KaiC proteins exhibit circadian cycles in abundance [30]–[33]. Moreover, overexpression of the *kaiC* gene for a few hours resets the phase of the rhythm [30], [33]. Experimentally however, the existing reports on transcriptional/translational *kaiBC* regulation (*transcriptional/translational feedback loop*, TTFL) are not consistent. For instance, several studies indicate that phospho-KaiC is mainly responsible for *kaiBC* suppression [34]–[37]. However, unphosphorylated KaiC has been shown convincingly to repress global transcription including its own upon overexpression [30], [32], [38]. Moreover, studies have implicated KaiA in the activation of *kaiBC* expression but only in cooperation with KaiC [30], [32]. The ATPase activity of KaiC is also suggested to drive transcription [39]. Taken together, these results have given rise to a model, wherein KaiC is proposed to function in the positive and in the negative limb of the *kaiBC* oscillatory loop. However, it is still not known which phospho-state of KaiC promotes and which phospho-state of KaiC suppresses expression of *kaiBC*.

In this work, we analyze various combinations of positive and negative regulation of *kaiBC* expression through KaiC by introducing a combined TTFL/PTO model that accounts for the different phospho-states of KaiC. Simulations of inactivation and overexpression of *kai* genes reveal that only one transcriptional feedback combination can reproduce the existing data satisfactorily. Importantly, the effects of simulated *kai*-knockout and *kai*-overexpression on *kaiBC* expression differ in the tested models depending on which phospho-form of KaiC drives *kaiBC* transcription and which phospho-form suppresses it.

## Results

### 12 possible two-loop transcriptional feedback models reproduce the observed dynamics of *kaiBC* expression and KaiC phosphorylation

For a theoretical investigation of which phospho-state of KaiC positively and which phospho-state of KaiC negatively regulates *kaiBC* transcription we chose existing *kaiBC* expression and KaiC phosphorylation data to state our constraints. We did image analysis of Figure S2 from Murayama et al. [35], where Northern and Western blot analyses were employed, to track the relative amount of *kaiBC* mRNA, unphosphorylated KaiC (UKaiC), and total phosporylated KaiC protein (PKaiC) in wild-type cells under constant light (LL) condition at 30°C. The levels of *kaiBC* mRNA, UKaiC and PKaiC were averaged and the ratios of UKaiC and PKaiC to total KaiC determined (Table S1). We chose the Murayama data because they provided time course data of *kaiBC* mRNA, UKaiC, and PKaiC protein levels from a single experiment. Here, each simulation was fit to the Murayama time course data resulting in optimal parameter sets (see Methods). The workflow was as follows: we analyzed whether the simulated peak phases of *kaiBC* mRNA, UKaiC and PKaiC protein levels gave good fits to the Murayama data and showed a period of ∼25 hours as observed experimentally [40]. If the period was about 24–26 hours but the simulated peak phases were not well reproduced we studied whether the simulation still can explain existing data on peak phases from other *in vivo* experiments [31], [40]–[42]. Provided the previous criteria were fulfilled, we tested further whether the model can also correctly reproduce the *kaiBC* mRNA expression dynamics observed in *kai* gene-knockout and overexpression mutants.

The model we developed couples our previous PTO model for the KaiABC core clock [16] to transcription/translation of the *kaiBC* operon resulting in a combined TTFL/PTO model. KaiC monomers are found in three different pools in the PTO portion of our model: KaiC monomers are part of a KaiC hexamer (**C** *^H^*-pool), a KaiBC complex (**C** *^B^*-pool) or are present in free monomers (**C** *^P^*-pool). In each pool, the KaiC monomers exist in four phosphorylation states U - unphosphorylated, T- threonine phosphorylated, S - serine phosphorylated and D - double phosphorylated. The production of new KaiC molecules occurs within the monomer pool. There, KaiC monomers assemble to hexamers to become active. For simplicity, all forms of KaiC are degraded with the same constant rate. Oscillation of *kaiBC* mRNA was realized by introducing a combination of a positive and a negative feedback loop into the model system. The element in the respective loop is KaiC. In the positive feed-forward loop, KaiC drives transcription of the *kaiBC* operon while in the negative feedback loop KaiC suppresses *kaiBC* transcription (see Methods and Text S1). We then studied the role of U-KaiC, T-KaiC, D-KaiC, S-KaiC, and total phosphorylated KaiC (P-KaiC) in *kaiBC* transcription with respect to positive and negative regulation. This kind of test is novel. In particular, we tested each phospho-form of KaiC within the **C** *^H^*-pool (*H^U+^*, *H^T+^*, *H^D+^*, *H^S+^*) as to positive *kaiBC* regulation. We disregarded phospho-forms of KaiC from the **C** *^B^*-pool because studies strongly indicate that they do not promote *kaiBC* expression [43], [44]. In addition, we considered each phospho-form of KaiC from the **C** *^H^*-pool (*H^U−^*, *H^T−^*, *H^D−^*, *H^S−^*) (Group I) and the **C** *^B^*-pool (*B^U−^*, *B^T−^*, *B^D−^*, *B^S−^*) (Group II) as to negative regulation of *kaiBC* (Table 1). For example, T-KaiC hexamers activate *kaiBC* transcription whereas U-KaiC hexamers inhibit it. We call this feedback combination the *H^T+^-H^U−^* model. Another example, T-KaiC hexamers activate *kaiBC* transcription whereas U-KaiBC complexes repress it. We call this feedback combination the *H^T+^-B^U−^* model.

**Table 1:** Overview of tested models.

GROUP I			GROUP II		
Hexamer pool (negative regulation)	Hexamer pool (positive regulation)	Figure	KaiBC complex pool (negative regulation)	Hexamer pool (positive regulation)	Figure
P-KaiC (*H^P−^*)	U-KaiC (*H^U+^*)	S2D	P-KaiC (*B^P−^*)	U-KaiC (*H^U+^*)	S4D
U-KaiC (*H^U−^*)	T-KaiC (*H^T+^*)	S2A	U-KaiC (*B^U−^*)	T-KaiC (*H^T+^*)	S4A
	D-KaiC (*H^D+^*)	S2B		D-KaiC (*H^D+^*)	S4B
	S-KaiC (*H^S+^*)	S2C		S-KaiC (*H^S+^*)	S5A
	TD-KaiC (*H^TD+^*)	1A		TD-KaiC (*H^TD+^*)	1D
	P-KaiC (*H^P+^*)	S1A		P-KaiC (*H^P+^*)	S4C
T-KaiC (*H^T−^*)	U-KaiC (*H^U+^*)	S1B	T-KaiC (*B^T−^*)	U-KaiC (*H^U+^*)	S5B
	D-KaiC (*H^D+^*)	1B		D-KaiC (*H^D+^*)	1C
	S-KaiC (*H^S+^*)	S3A		S-KaiC (*H^S+^*)	S5C
D-KaiC (*H^D−^*)	U-KaiC (*H^U+^*)	S3B	D-KaiC (*B^D−^*)	U-KaiC (*H^U+^*)	S4E
	T-KaiC (*H^T+^*)	S3C		T-KaiC (*H^T+^*)	S5D
	S-KaiC (*H^S+^*)	S1C		S-KaiC (*H^S+^*)	S5E
S-KaiC (*H^S−^*)	U-KaiC (*H^U+^*)	S3D	S-KaiC (*B^S−^*)	U-KaiC (*H^U+^*)	S6A
	T-KaiC (*H^T+^*)	S1D		T-KaiC (*H^T+^*)	S6B
	D-KaiC (*H^D+^*)	S3E		D-KaiC (*H^D+^*)	S6C
	TD-KaiC (*H^TD+^*)	S1E		TD-KaiC (*H^TD+^*)	S6D

One can argue (1) that D-KaiC follows T-KaiC close in time and thereby it would be hard to dissect the single contribution of both phospho-forms of KaiC on *kaiBC* transcription or (2) that all three phosphorylated forms of KaiC (T-KaiC, D-KaiC, S-KaiC) may act on the *kaiBC* promoter. Therefore, we also took into consideration that T-KaiC and D-KaiC (*H^TD+^*) as well as T-KaiC, D-KaiC, and S-KaiC (*H^P+^*) from the **C** *^H^*-pool compete for the *kaiBC* promoter. Furthermore, we considered that T-KaiC, D-KaiC and S-KaiC from the **C** *^H^*- and the **C** *^B^*-pool compete for the *kaiBC* promoter to inactivate transcription (*H^P−^* and *B^P−^*, respectively). Although regulation of *kaiBC* could also be *via* heterogenous KaiC hexamers states we show with a binomial distribution calculation that using the homogenous phospho-states U, T, D and S as responsible for the feedback regulation is a reasonable assumption (see Text S1).

In the end, we tested 32 combinations (Table 1). Optimal parameters for each model were identified (Table S3, see also Methods). We deliberately based our models exclusively on the cycling dynamics of the four KaiC forms to test whether we still could arrive at an output that is congruent with the experimental data. In particular, we disregarded other clock-related proteins that might be involved in transcriptional regulation [34].

**Figure 1:**
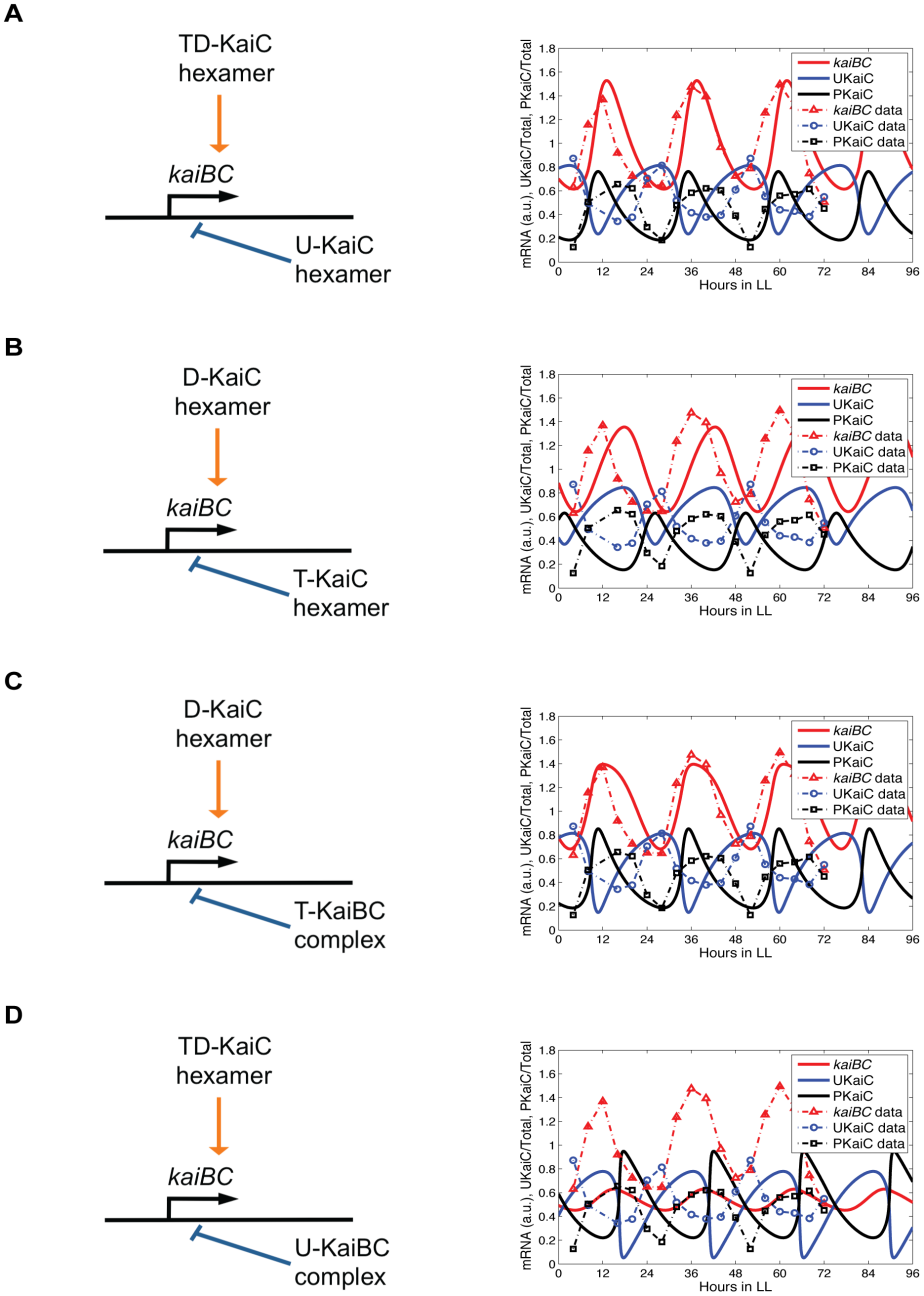
Simulations of models with different combinations of positive and negative transcriptional feedback regulation of the *kaiBC* operon. 12 of 32 tested two-loop feedback models, each six of Group I and Group II, sufficiently reproduce the experimental observed phase relations between *kaiBC* mRNA, unphosphorylated KaiC (UKaiC) and total phosphorylated KaiC (PKaiC) protein and period of oscillation. In Group I and Group II, the transcriptional repressor originates from the hexamer pool (**C** *^H^*-pool) and the KaiBC complex pool, respectively (**C** *^B^*-pool; see also Table 1). (**A and B**) Representative time-series of a good-fit (**A**) and a not-good-fit model (**B**) of Group I: the *H^TD+^*-*H^U−^* and the *H^D+^*-*H^T−^* model. (**C and D**) Representative time-series of a good-fit (**C**) and a not-good-fit model (**D**) of Group II: the *H^D+^*-*B^T−^* and the *H^TD+^*-*B^U−^* model. As examples, *H^D+^*, double phosphorylated KaiC (D-KaiC) from the **C** *^H^*-pool promotes *kaiBC* transcription; *H^T−^*, threonine phosphorylated KaiC (T-KaiC) from the **C** *^H^*-pool suppresses *kaiBC* transcription; *B^T−^*, threonine phosphorylated KaiC (T-KaiC) from the **C** *^B^*-pool suppresses *kaiBC* transcription. Fitted oscillations of *kaiBC* mRNA, UKaiC, and PKaiC protein levels are shown as red, blue and black solid curves, respectively. The average level of *kaiBC* transcription was standardized to 1. The levels UKaiC und PKaiC are ratios to total KaiC. The symbols represent data from image analysis (see Methods; Table S1). The results of the other model fits are summarized in Figures S1, S2, S3 (Group I models) and Figures S4, S5, S6 (Group II models). The parameters are given in Table S3. The subjective-day phase is from 0 to 12 hours (LL0-12), the subjective-night phase from 12 to 24 hours (LL12-24).

Six models in each of both two-loop feedback network groups reproduce the observed dynamics of *kaiBC* expression and KaiC phosphorylation. The most promising models of Group I, in which each phospho-form of KaiC from the **C** *^H^*-pool negatively feeds back on *kaiBC* transcription, are the following: two models in which U-KaiC hexamers repress *kaiBC* transcription and TD-KaiC hexamers or all three phosphorylated forms of KaiC promote it (*H^TD+^*-*H^U−^*; *H^P+^*-*H^U−^*); one model in which T-KaiC hexamers downregulate *kaiBC* transcription and U-KaiC hexamers activate the *kaiBC* promoter activity (*H^U+^*-*H^T−^*); one model in which D-KaiC hexamers repress *kaiBC* transcription and S-KaiC hexamers turn *kaiBC* transcription on (*H^S+^*-*H^D−^*); and two models in which S-KaiC hexamers suppress *kaiBC* transcription and T-KaiC hexamers or TD-KaiC hexamers promote it (*H^T+^*-*H^S−^*; *H^TD+^*-*H^S−^*). Figure 1A shows a simulated expression profile of the *H^TD+^*-*H^U^* model as an example of a good fit model of Group I. The results from the other five data fits are given in Figure S1. In summary, *kaiBC* mRNA oscillates with maximal expression 6–13 h after dawn, UKaiC cycles with peak phases during the first half of the subjective day (LL0-7) whereas maximal KaiC phosphorylation occurs from LL7 to LL15 as observed experimentally [31], [40]–[42]. The oscillations consistently follow a period of 24–26 h in LL (Table S2). Other tested feedback combinations of Group I cannot explain the data points satisfactorily despite extensive parameter space searches. A prime example of a model which deviate from experiments is shown in Figure 1B. The full results are summarized in Figure S2 and S3 (see also Table S2).

Six simulations of feedback combinations of Group II also explain the peak phases of *kaiBC* mRNA, UKaiC and PKaiC levels under LL condition, showcased for the *H^D+^*-B*^T−^* model in Figure 1C. In the Group II, phospho-forms of KaiC from the **C** *^B^*-pool negatively feed back on *kaiBC* transcription. Further good fit models are *H^T+^*-*B^U−^*, *H^D+^*-*B^U−^*, *H^P+^*-*B^U−^*, *H^U+^*-*B^P−^*, and *H^U+^*-*B^D−^* (Figure S4). Other transcriptional feedback combination cannot recapitulate the expression dynamics as observed experimentally (Figure S5 and S6). An example expression profile is shown in Figure 1D.

### Three models correctly reflect the *kaiBC* expression and phosphorylation dynamics in the *kaiA* mutant

Of 32 tested combinations for *kaiBC* feedback regulation, 12 generated time courses fit to existing experimental data. Six models in which in each case the negative KaiC feedback species originates from the **C** *^H^*-pool (Group I) and six models in which in each case the negative KaiC feedback species is from the **C** *^B^*-pool (Group II). In a next step we tested whether these models would hold true if we simulate nullification of the *kaiA* gene as was done by setting the *kaiA* transcription rate to zero (Text S1). From previous experiments we know that *kaiA*-inactivated (*kaiA* ^−^) strains reduce *kaiBC* promoter activity relative to the wild type [30], [32]. Additionally, the lack of the KaiA protein causes the unphosphorylated form of KaiC (U-KaiC) to be most abundant in the cell [32]. This suggests U-KaiC states to inhibit *kaiBC* transcription. On the other hand, Murayama et al. plausibly show that phosphorylated KaiC forms mainly regulate repression of the *kaiBC* promoter activity [35]. Therefore, we deliberately decided not to impose any constraints as to which phospho-state of KaiC promotes and suppresses, respectively, *kaiBC* transcription and analyzed the good-fit models further.

**Figure 2:**
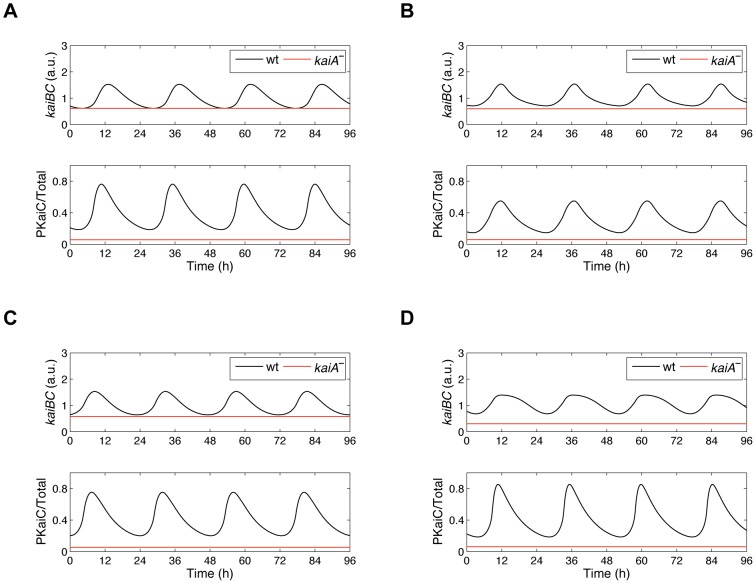
Four two-loop feedback models reproduce the effects of *kaiA* knockout mutants on *kaiBC* expression and KaiC phosphorylation. Predicted time-series of *kaiBC* expression and KaiC phosphorylation in the absence of the *kaiA* gene. Deletion of the *kaiA* gene was simulated through setting the *kaiA* transcription rate to zero. Of the six models of Group I, which captured the measured *kaiBC* expression and KaiC phosphorylation dynamics, three models correctly reflect the effects of *kaiA* depletion as well: *H^TD+^*-*H^U−^* (**A**), *H^P+^*-*H^U−^* (**B**), and *H^TD+^*-*H^S−^* (**C**). Simulated deletion of *kaiA* transcription in these models destroys *kaiBC* gene expression and KaiC phosphorylation rhythm in parallel. The levels of *kaiBC* mRNA and PKaiC are reduced. These models were analyzed further in Figure 4. (**D**) Of the six models of Group II, only the *H^D+^*-*B^T−^* model correctly reflects the effects of *kaiA* depletion as well. This model, however, cannot reproduce upregulation of *kaiBC* expression upon overexpression of the *kaiA* gene (see Figure S9). The *H^U+^*-*H^T−^*, *H^S+^*-*H^D−^* and *H^T+^*-*H^S−^* models of Group I and the *H^T+^*-*B^U−^*, *H^D+^*-*B^U−^*, *H^P+^*-*B^U−^*, *H^U+^*-*B^P−^*, and *H^U+^*-*B^D−^* fail to recapitulate downregulation of *kaiBC* expression upon *kaiA* inactivation (Figure S7). The abbreviations are explained in Figure 1.

**Figure 3:**
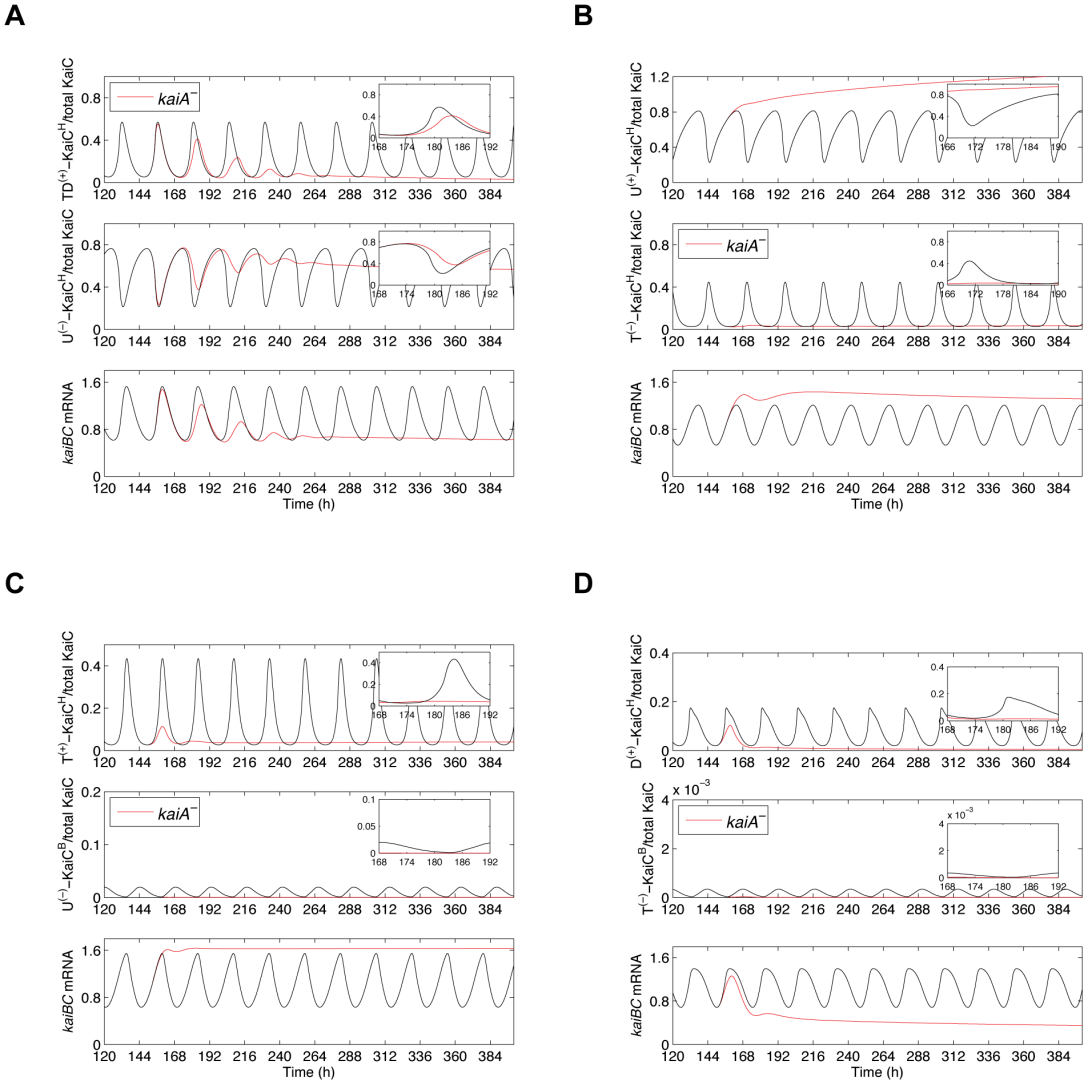
Initial dynamics of the transcriptional KaiC feed-back species in simulated *kaiA* knockout mutants. Each panel depicts the simulated expression dynamics of the positive transcriptional regulator, the negative transcriptional regulator and *kaiBC* mRNA for the first days in LL after *kaiA* transcription was removed. (**A and B**) Predicted time-series for two models of Group I. The *H^TD+^*-*H^U−^* model (**A**) predicts decreased *kaiBC* mRNA levels in the absence of *kaiA* transcription. In this simulation, TD-KaiC phosphorylation ceases and U-KaiC constitutively accumulates. As a result, *kaiBC* transcription is suppressed. Down-regulation of *kaiBC* was predicted from all models in which U-KaiC hexamers are assumed to suppress *kaiBC*. The *H^U+^*-*H^T−^* model (**B**) predicts an enhanced *kaiBC* level when the *kaiA* gene is absent. In this *kaiA*-knockout simulation the threonine phosphorylated KaiC hexamer level drops immediately. There are no T-KaiC hexamers, which could negatively feed back on *kaiBC*. In addition, U-KaiC hexamers increase steadily. As a result, *kaiBC* expression is not reduced. All models in which D-KaiC, T-KaiC, and S-KaiC hexamers negatively feed back on *kaiBC* cannot reproduce suppression of *kaiBC* when *kaiA* is absent. After removing *kaiA* transcription KaiC phosphorylation ceases abruptly such that the negative feedback loop is not functional to down-regulate *kaiBC* transcription. However, three exceptions suggest that the peak amplitude rhythms of the transcriptional activator and the transcriptional repressor species are crucial (Figure S8). (**C and D**) Predicted time-series for two models of Group II. The peak amplitude rhythms of the U-KaiBC complexes in the *H^T+^*-*B^U−^* model (**C**) are too low to fulfill the role as negative regulator of *kaiBC* transcription in the *kaiA^−^* mutant. Only the enhanced retention of the transcriptional activator (D-KaiC hexamers) in the *H^D+^*-*B^T−^* model (**D**) alone can suppress *kaiBC* expression rhythm in the simulated *kaiA*-knockout mutant. Note the different Y-scalings. The abbreviations are explained in Figure 1.

In all 12 tested models, *kaiA* deletion abolishes overt circadian rhythms of *kaiBC* mRNA and PKaiC. Furthermore, KaiC phosphorylation reaches consistently a constant minimum of ∼0% phosphorylated KaiC (Figure 2 and S7). However, deletion of the *kaiA* gene reduces the *kaiBC* mRNA level only in the *H^TD+^*-*H^U−^*, *H^P+^*-*H^U−^* and *H^TD+^*-*H^S−^* models of Group I (Figure 2A–C) as well as in the *H^D+^*-*B^T−^* model of Group II (Figure 2D). By contrast, the absence of the *kaiA* gene in the other eight models leads to higher *kaiBC* expression levels, which contradict the observed positive role of KaiA on *kaiBC* (Figure S7). It implies that KaiA has lost its positive influence on *kaiBC* expression. We hypothesized that is due to a dysfunctional negative feedback loop in these models. In order to investigate this hypothesis, we studied the dynamics of the respective positive and negative KaiC feedback species in all 32 tested models shortly after *kaiA* transcription has been removed. Figure 3 gives two representative simulation results of Group I and Group II showing the dynamics of *kaiBC* expression and of the KaiC phospho-forms which feed forward and back, respectively, on *kaiBC*. *kaiA* transcription was removed by the time *kaiBC* transcription had achieved its minimum (Text S1). As seen for the *H^TD+^*-*H^U−^* model, oscillation of TD-KaiC hexamers damps out as U-KaiC hexamers do (Figure 3A). In agreement with existing experiments, the levels of *kaiBC* mRNA and KaiC phosphorylation are constitutively reduced whereas the amount of U-KaiC hexamers is enhanced. An explanation for these damped oscillations is as follows: In the first cycle, the quantities of T-KaiC and D-KaiC hexamers suffice to promote *kaiBC* expression. Newly synthesized KaiC proteins are phosphorylated very fast. Repression of *kaiBC* transcription is low due to a small quantity of U-KaiC hexamers. As the levels of T-KaiC and D-KaiC hexamers reach their peak, degradation takes over the dynamics such that T-KaiC and D-KaiC hexamer levels drop resulting in suppression of *kaiBC* by U-KaiC hexamers. With lacking KaiA proteins, TD-KaiC phosphorylation ceases and U-KaiC constitutively accumulates to repress further transcription of *kaiBC*. These dynamics were observed in those models in which U-KaiC hexamers are assumed to suppress *kaiBC*. By contrast, the *H^U+^*-*H^T−^* model does not show such a behavior (Figure 3B). Rather, the level of threonine phosphorylated KaiC hexamers drops immediately. There are not any T-KaiC hexamers, which could negatively feed back on *kaiBC*. In addition, U-KaiC hexamers increase steadily. As a result, *kaiBC* expression is not reduced. Interestingly, each tested model in which T-KaiC, D-KaiC and S-KaiC hexamers is assumed to inhibit *kaiBC* transcription could not replicate the downregulation of *kaiBC* as seen in *kaiA^−^*-mutant strains. After removing *kaiA* transcription KaiC phosphorylation ceases abruptly such that the negative feedback loop is not functional to suppress *kaiBC* transcription. However, three models suggest that suppression of *kaiBC* is possible if there is a proper abundance ratio of the transcriptional activator to repressor (Figure S8A). Thus, removing the *kaiA* gene from the *H^T+^*-*H^D−^* model turns *kaiBC* transcription down as well. Here, D-KaiC hexamers (negative regulator) display a lower expression rhythm than T-KaiC hexamers (positive regulator) but the oscillation damps out more slowly than that of T-KaiC hexamers such that the negative feedback loop is functional to suppress *kaiBC* transcription further. In the *H^TD+^*-*H^S−^* and *H^D+^*-*H^S−^* models D-KaiC and S-KaiC hexamers display nearly the same peak expression rhythm shortly after *kaiA* has been removed but the level of S-KaiC hexamers (negative regulator) again damps out more slowly. This causes constitutive suppression of *kaiBC* (Figure S8B, C).

In the case of the Group II models, where in each combination of positive and negative regulation the transcriptional repressor is from the KaiBC complex pool, we reason that the peak expression rhythms of KaiBC complexes are always too low to fulfill the role as negative regulator of *kaiBC* transcription in the *kaiA^−^* mutant (Figure 3C). Only the enhanced retention of the transcriptional activator alone can suppress *kaiBC* expression rhythm in the simulated *kaiA*-knockout mutant (Figure 2D, 3D). This retention is also the reason why simulated *kaiA*-overexpression causes decreased *kaiBC* transcript levels as well as observed for the *H^D+^*-*B^T−^* model contradicting experimental findings (Figure S9). In summary, we rejected the idea that phospho-forms of KaiC from the **C** *^B^*-pool function as transcriptional repressors and decided to analyze the *H^TD+^*-*H^U−^*, *H^P+^*-*H^U−^* and *H^TD+^*-*H^S−^* model in more detail.

### The *H^TD+^*-*H^U−^* model reproduces the *kaiBC* expression dynamics of ox*kaiA* and ox*KaiC* mutants

**Figure 4:**
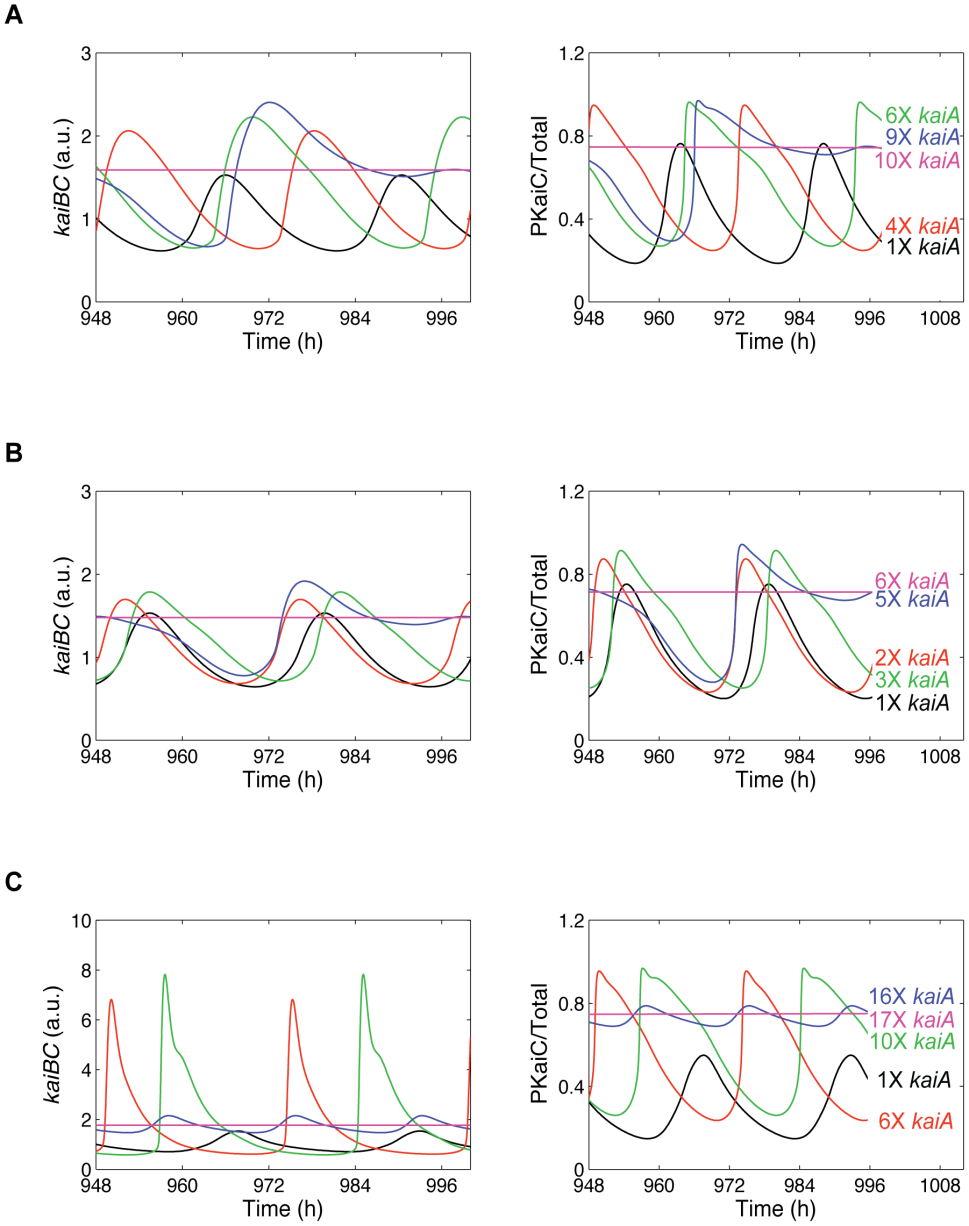
Sensitivity of *kaiBC* mRNA and KaiC phosphorylation dynamics against stepwise increase in KaiA protein. Shown are simulations for the *H^TD+^*-*H^U−^* model (**A**), the *H^TD+^*-*H^S−^* model (**B**), and the *H^P+^*-*H^U−^* model (**C**). Enhanced concentration of the *kaiA* transcript and thus KaiA protein was simulated through enhancing the transcriptional rate of the *kaiA* gene. The three models show different sensitivity against changes in the *kaiA*-transcriptional rate. The models in (**A**) and (**B**) were analyzed further in Figure 5. The abbreviations are explained in Figure 1.

Several *kaiA* overexpression (ox*kaiA*) studies showed that KaiC becomes progressively more hyper-phosphorylated meaning in particular mainly threonine and double phosphorylated forms of KaiC accumulate and become constant in time [30], [37], [41]. In agreement with these observations, our published PTO model, which is part of our combined TTFL/PTO model in this study, also correctly simulates a higher KaiC phosphorylation level when KaiA is solely enhanced [16]. Additionally, elevated KaiA levels dose-dependently increase *kaiBC* expression and damp it to arhythmicity [30], [37], [41]. Thus, repression of the KaiC phosphorylation rhythm correlates with the suppression of the *kaiBC* transcription rhythm as simulated by the three remaining models (*H^TD+^*-*H^U−^*, *H^P+^*-*H^U−^*, *H^TD+^*-*H^S−^*) of our analysis as well (Figure 4). In all three models, threonine and double phosphorylated KaiC hexamers compete for the *kaiBC* promoter to activate transcription. Consequently, we would expect that these models reproduce the same *kaiBC* expression dynamics upon an excess of KaiA proteins. The simulation results show that in the *H^TD+^*-*H^U−^* model and in the *H^TD+^*-*H^S−^* model *kaiBC* mRNA and KaiC phosphorylation rhythm were consistently suppressed with a 6–10-fold higher transcriptional activity of *kaiA* (Figure 4A, B). Note the transcriptional activators are identical in both models, only the repressor with U-KaiC hexamer and S-KaiC hexamer, respectively, is different. At this point in our analysis we asked whether S-KaiC and U-KaiC hexamers compete for the *kaiBC* promoter and thus suppress *kaiBC* transcription. However, such a feedback combination could not reproduce the peak phase of *kaiBC* mRNA and a rhythm of 24 hours (Figure S10; Table S2).

Surprisingly, the *H^P+^*-*H^U−^* model simulates a different dynamical behavior of accumulation of *kaiBC* transcripts although there is not much difference between the *H^TD+^*-*H^U−^* and *H^P+^*-*H^U−^* models. The sole difference is that serine phosphorylated KaiC hexamers in addition T-KaiC and D-KaiC hexamers can promote *kaiBC* transcription in the *H^P+^*-*H^U−^* model. However, a 17-fold increase in *kaiA* transcription is required to finally eliminate any rhythm in the *H^P+^*-*H^U−^* model (Figure 4C) that is in contrast to simulations of the *H^TD+^*-*H^U−^* and *H^TD+^*-*H^S−^* models. Furthermore, up to a 16-fold value, the *kaiBC* amplitude and KaiC phosphorylation rhythm strongly increase in order to then abruptly decreases. Such an abrupt dynamical behavior is not observed in both *in vitro* and *in vivo* experiments. We therefore could reject another combination of transcriptional feedback regulation [37], [41], [45].

**Figure 5:**
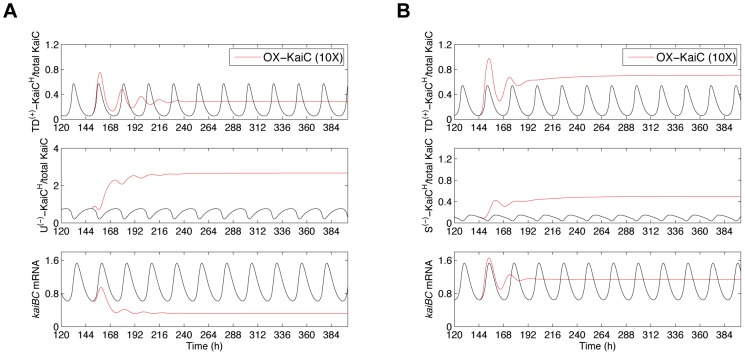
Initial dynamics of the transcriptional KaiC feed-back species in simulated KaiC-overexpression mutants. KaiC overexpression was simulated through increasing the translational rate of unphosphorylated KaiC monomers at time of minimal *kaiBC* expression. Each panel depicts the simulated expression dynamics of the positive transcriptional regulator, the negative transcriptional regulator and *kaiBC* mRNA for the first days in LL after *KaiC-*overexpression was induced in the (**A**) *H^TD+^*-*H^U−^* and (**B**) *H^TD+^*-*H^S−^* models. The *H^TD+^*-*H^U−^* feedback model reproduces the effects of KaiC overexpression on *kaiBC* transcription. The abbreviations are explained in Figure 1.

In a next step we asked whether we could rule out one of the two remaining feedback mechanisms by simulating constitutive overexpression of KaiC. We followed a previous lab experiment where a reporter strain was transformed with plasmid pTS2KP*_trc_*::*kaiC* to ectopically induce overexpression of the *kaiC* gene [32]. Here, we simulated constitutive overexpression of *kaiC* in both models by increasing the translational rate of unphosphorylated KaiC monomers at the time of minimal *kaiBC* expression (see Text S1). In the *H^TD+^*-*H^U−^* model, KaiC phosphorylation and UKaiC expression rhythms damp out (Figure 5A). UKaiC hexamers consistently exist in large excess that results in suppression of *kaiBC* [32]. Elevated levels of U-KaiC cease any rhythm in the *H^TD+^*-*H^S−^* model as well (Figure 5B). In this case, however, the positive transcriptional regulators (T-KaiC and D-KaiC hexamers) are more abundant than the repressor (S-KaiC hexamers). This means that positive regulation of *kaiBC* transcription outweigh negative regulation. Therefore, a complete suppression of *kaiBC* is not possible.

**Figure 6:**
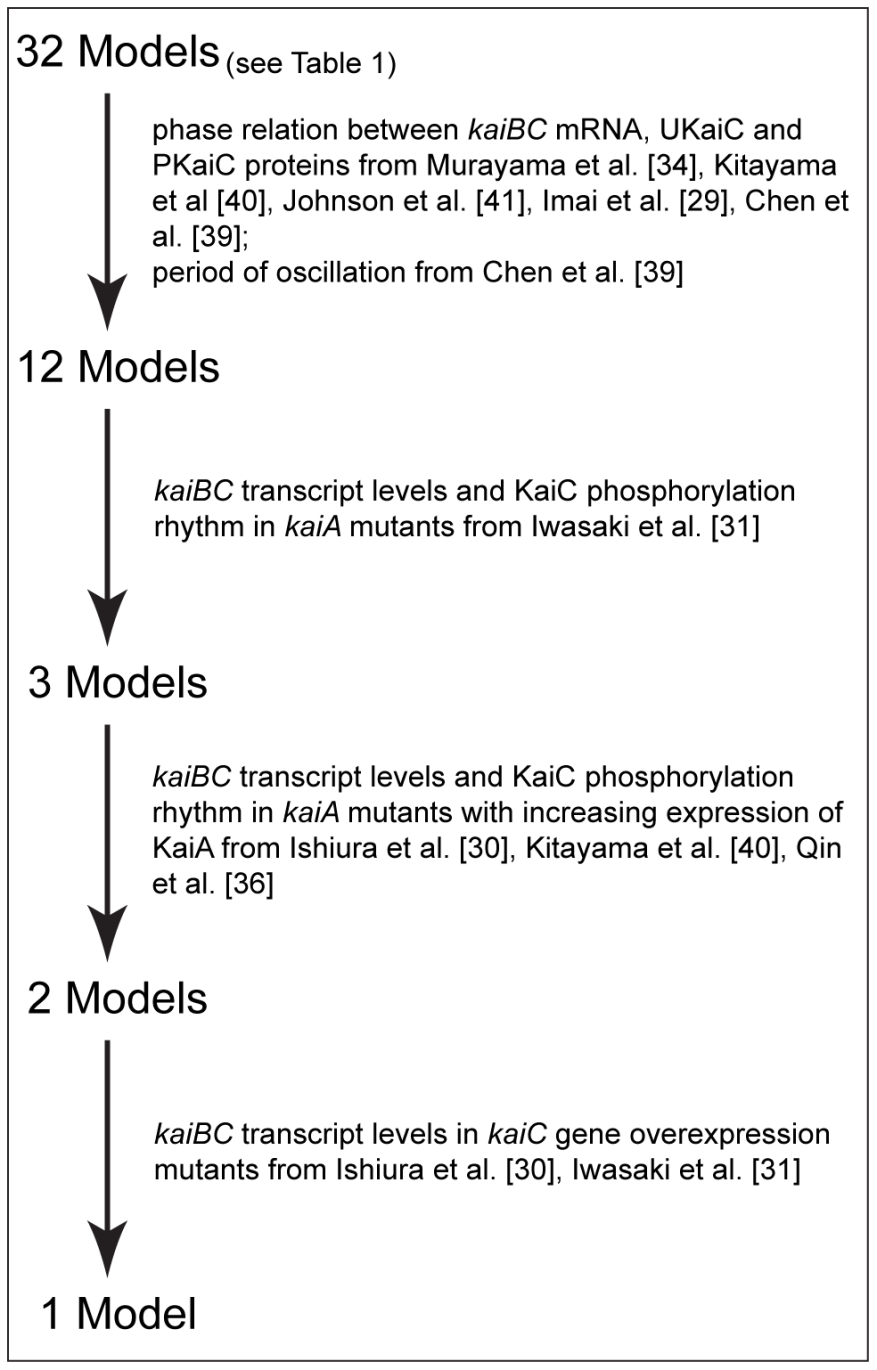
Workflow of the model selection process.

In the *H^TD+^*-*H^U−^* model, U-KaiC hexamers are assumed to suppress *kaiBC* transcription. To exclude that simulated downregulation of *kaiBC* is only due to the assumed negative feedback control we also simulated the ox*KaiC* mutant for the *H^U+^*-*H^T−^* and *H^S+^*-*H^D−^* models from Figures S1B and S1C. Note that these two models did not capture the effects of *kaiA* deletion. We found however that both feedback models caused suppression of *kaiBC* transcription in response to induced overexpression of U-KaiC monomers (Figure S11). We could thus obviate that our assumption in the *H^TD+^*-*H^U−^* model, namely that U-KaiC hexamers suppress *kaiBC*, implied the reduced *kaiBC* mRNA levels in the simulated ox*KaiC* mutant. Rather, we reason that again the peak expression ratio of the transcriptional activator to repressor determines the effect of induced KaiC overproduction on *kaiBC*. To sum up, from our 32 tested combinations of positive and negative regulation of *kaiBC* transcription *via* the four phospho-states of KaiC, only a particular two-loop feedback mechanism has remained (see also Figure 6).

## Discussion

Existing data support the view that the different phospho-states of KaiC govern the timing mechanism of the cyanobacterial circadian oscillator as well as clock output generating 24 h gene expression rhythms. In addition, KaiC was shown to promote expression of its own *kaiBC* transcript and to repress it. However, which phospho-state of KaiC is involved in transcriptional activation and which in transcriptional suppression has remained unclear due to inconsistent reports [30], [34]–[36]. In this study, we developed a combined TTFL/PTO model, which considers stepwise KaiC phosphorylation and dephosphorylation. Using the combined TTFL/PTO model we investigated which phospho-states of KaiC are positive and negative elements of *kaiBC* expression by analyzing systematically various combinations of transcriptional feedback regulation – 32 in this study. We found for many tested models that when the expression level of the transcriptional repressor is too low compared to the level of the activator, positive regulation outcompetes negative regulation. This can be particularly seen in those two-loop feedback combinations, in which different phospho-states of KaiBC complexes negatively feed back on *kaiBC* (Figures 3C, S9). Interestingly, our simulations showed that only a particular combination of positive and negative feedback loops could reproduce the observed dynamics of *kaiBC* expression and the KaiC phosphorylation cycle, including the phenotypes of *kaiA* gene-knockouts and KaiA and KaiC overexpressors. *In vitro* experiments show that KaiC phosphorylation does not depend on variations of KaiB protein, provided that a minimal amount of KaiB protein is present [17], [46]. We conclude that variations of *kaiB* transcription rates have no effect on KaiC phosphorylation in the *in vivo* system. We, therefore, have focused on overexpression studies of KaiA and KaiC.

Thus, we propose that threonine and double phosphorylated KaiC hexamers promote *kaiBC* transcription whereas the unphosphorylated KaiC hexamers shut it off. Our suggested two-loop feedback model is in perfect agreement with experiments, in which overexpression of U-KaiC represses its own transcription [30], [38]. Further, our suggestion that T-KaiC and D-KaiC hexamers promote transcription of *kaiBC* agrees a study in which peak KaiC phosphorylation and ATPase activity are closely coupled and thought to trigger the activation of *kaiBC* expression [39]. Peak KaiC ATPase activity occurs towards the end of the subjective day *in vivo* and may dictate the timing of KaiC phosphorylation [39]. We are aware of published data, which indicate that U-KaiC hexamers release phosphorylated SasA at dawn which in turn transfers its phosphate group to RpaA [44]. This in fact would mean that U-KaiC hexamers indirectly promote expression of *kaiBC*. However, our tested models where U-KaiC hexamers are assumed to turn *kaiBC* transcription on (*H^U+^*-*H^T−^*, *H^U+^*-*H^D−^*, *H^U+^*-*H^S−^* and *H^U+^*-*H^P−^*) failed to reproduce suppression of *kaiBC* when the *kaiA* gene is absent.

**Figure 7:**
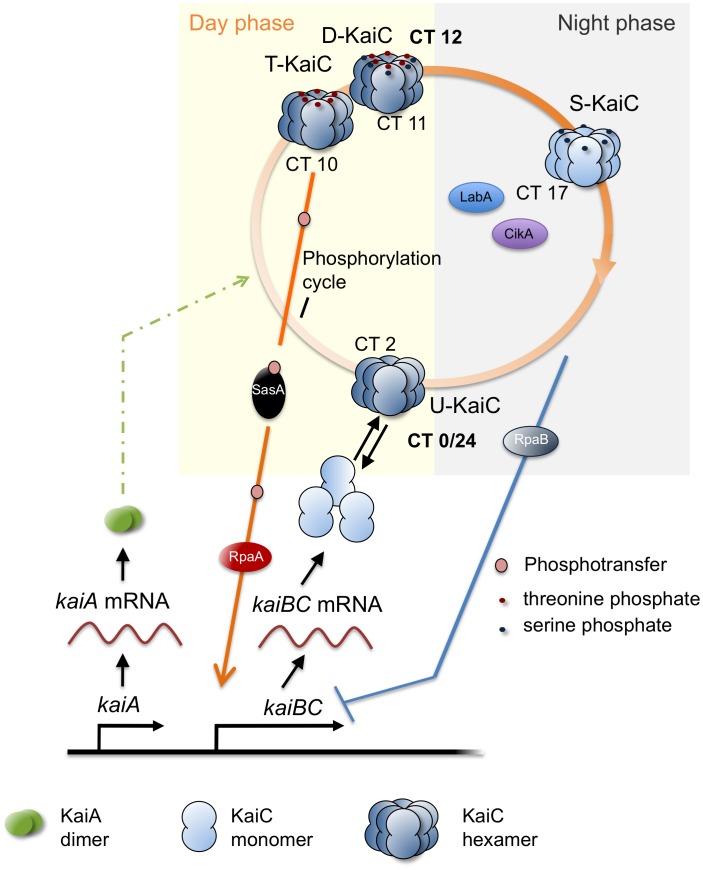
The *H^TD+^*-*H^U−^*-two-loop feedback model for the cyanobacterial circadian clock. KaiB translation was not considered in the model because KaiB has only little effect on the autophosphatase activity of KaiC at 30°C [7], [46], [58]. Therefore, KaiB is omitted from the figure. Details are described in the text.

The picture of circadian regulation of *kaiBC* transcription that emerges from our theoretical analysis is as follows (Figure 7): Depending on its phospho-state, KaiC activates and represses clock-related proteins, which regulate the transcription of many clock target genes, including the *kaiBC* gene cluster itself. For example, SasA and RpaA function in the daytime positive feedback loop. By contrast, CikA, LabA, and RpaB are negative elements of the nighttime pathway. During the first half of the night, LabA and CikA likely initiate repression of the activity of RpaA through interaction with inhibitory proteinaceous factors so that transcription of *kaiBC* starts to decline [22], [34]. Later in the night phase, an additional transcriptional regulator accumulates, RpaB. Since the unphosphorylated KaiC hexamers are most prevalent at that time as well, we propose that the KaiC hexamers signal their unphosphorylated state through an so far unknown mechanism, so that RpaB becomes active and binds specifically to the *kaiBC* promoter as shown experimentally [22]. Consequently, transcription of *kaiBC* is suppressed permanently. At this point, unphosphorylated KaiC hexamers may set in train a series of events. They exist in abundance and interact with a delay with KaiA. KaiA has a high affinity to U-KaiC hexamers. Complementarily, U-KaiC hexamers may also trigger dephosphorylation of SasA. Thus during daytime, U-KaiC hexamers become less abundant because KaiA promotes autophosphorylation of KaiC. The next circadian cycle is initiated in which T-KaiC and D-KaiC hexamers activate the positive limb of the *kaiBC* oscillatory loop. Experimentally, it is shown that phosphorylation of KaiC and SasA-RpaA peak from subjective day to dusk under constant light (LL) conditions (from LL8 to LL16) [19], [31], [41], [42]. At that time, SasA very likely interacts with the T-KaiC and D-KaiC hexamers and thereby mediates a phospho-transfer to RpaA. We follow the suggestion by Hanaoka et al. [22] that RpaA may mediate the dissociation of RpaB from the *kaiBC* promoter region and the *kaiBC* operon is transcribed. In summary, the competing actions of ‘positive’ (TD-KaiC hexamers, SasA, RpaA) and ‘negative’ factors (U-KaiC hexamers, LabA, CikA, RpaB) are separated in time. Furthermore, the two actions initiate each other. So far, a further positive-negative feedback loop, coupled or uncoupled from the core clock, has not been reported for other genes in cyanobacteria. Though, an alternative two-loop regulation of gene expression is known for the light-responsive gene *psbA* with, separated in time, sigma factor-mediated positive and negative regulation for the transcriptional and post-transcriptional step of *psbA* expression, respectively [47].

On the other hand, there are genes, which resemble the *kaiBC* gene cluster in high amplitude and peak time of expression rhythm, such as the circadian input histidine kinase gene *cikA* and the circadian response regulator gene *rpaA* as well as transcripts of three sigma factor genes, *rpoD5/sigC*, *rpoD6* and *sigF2* [48]. Previous studies have already suggested sigma factors to be involved in the circadian output control as well [49], [50]. Thus, activation and repression of *kaiBC* expression is accompanied by transcriptional activation and inhibition of many clock-related genes. In *Synechococcus*, about 30% [48] to 64% [27] of the entire transcriptome is under circadian control. The output pathways for *kaiBC* expression are likely required for the clock machinery to coordinate circadian gene expression globally, through basic transcriptional activity and changes in the chromosome status, which in turn affect transcriptional rates [26]. The interplay of local and global transcription control may explain the variety of amplitude and phase rhythms of circadian promoter activities [48], [51].

Similar two-loop feedback structures are found in the clock of fungi [52], flies [53] and mammals [54]. Furthermore, results strongly indicate that positive and negative feedbacks together sustain the amplitude of circadian gene expression rhythms [55]. In these species, key transcriptional factors, such as fungal Frequency (FRQ), fly Period (PER) and Timeless (TIM), and mouse mPER and mCRY, have two functions. For example, mouse BMAL1 drives rhythmic clock gene expression through its association with its constitutively available partner, CLOCK. The logical equivalent of BMAl1 and CLOCK in the cyanobacteria clock system could be TD-KaiC and KaiA, respectively. Furthermore, similar to cyano U-KaiC, mouse mCRY and mPER are known to suppress its own expression by turning off its mBMAL1-mCLOCK-dependent transcription. In their second role, elevated levels of mPER and mCRY in the current cycle stimulate transcription of *mBMAL1* for the next. In the cyanobacteria system, the abundance of U-KaiC leads to KaiC autophosphorylation promoted by KaiA.

Another similar mechanism is found in the mouse system where RORα and REV-ERBα regulate transcription of their target genes, which include themselves by promoting and repressing, respectively, transcription of BMAL1 [53], [54], [55]. Outside but linked to the two-core loop as well are the clock proteins E4BP4 and DBP. E4BP4 is indirectly activated by the BMAL1-CLOCK dimer and suppressed by mPER and mCRY, as is the case with the *dbp* gene. In this case, DBP activates whereas E4BP4 suppresses the transcription of clock target genes at different times of day [56] that is analogous to cyano RpaA and cyano RpaB, respectively. Thus, despite the differences in detail, the various mammalian factors seem to interact within interlocked positive and negative loops that are functionally comparable to those of cyanobacteria.

Based on the work of Bintu et al. [57], we chose a minimal set of parameters, which regulates transcription of *Synechococcus kaiBC*. Thus, the *kaiBC* gene expression is assumed to be dependent only on the concentration of each phospho-state of KaiC. Interactions of KaiC with other clock-related transcription factors (e.g. SasA/RpaA, RpaB), regulating *kaiBC* transcription, are lumped into two effective regulation factors, which describe the fold-change in *kaiBC* gene expression approximately. In doing so, we assume simple activation and simple repression for the regulation of transcription of the *kaiBC* operon. Furthermore, using Bintu et al.'s thermodynamic model of gene regulation, we also assume that transcription initiation is proportional to the steady-state level of expression of the *kaiBC* gene. However, the difficulty of this simplification lies in the fact that there are very likely several different mechanisms that can interfere with the expression of *kaiBC* and thus also affect the response to overexpression and deletion of *kai* genes, such as transcriptional and/or posttranscriptional modification mechanisms. Besides, we did not consider the contribution of several different mechanisms to *kaiBC* expression (e.g. noncircadian regulation, cooperative interaction with KaiC ATPase). Consequently, we cannot completely rule out that other combinations of positive and negative feedback loops reflect the regulation of *kaiBC* expression in the living cell more reliably. However, using our combined TTFL/PTO model systems, we analyzed as many reasonable combinations of positive and negative regulation of *kaiBC* transcription as possible and provided for each model the optimal values of the respective parameters, which can be used for further theoretical studies (Table S3). As more experimental data become available, it will be possible to re-evaluate our proposed two-loop feedback model as to whether it can still consistently explain the experimental data. In the case, where this model is found wanting, it can be extended with, for example, other regulatory loops of the clock input/output. Alternatively, the other 31 tested models could be re-examined. Finally, our TTFL/PTO model system with its various combinations of positive and negative transcriptional feedback regulation together with future advances in experiments could help to reveal how the circadian output pathways allow the KaiC protein to control several hundred rhythmically regulated genes in the cyanobacterial genome.

## Methods

Our mathematical model comprises a post-translational oscillator (PTO) and a transcriptional/translational feedback loop (TTFL). The PTO is based on rhythmic KaiC phosphorylation and is described in detail by Brettschneider et al. [16]. Briefly, the KaiC monomers in the PTO portion are part of a KaiC hexamer (**C** *^H^*-pool), a KaiBC complex (**C** *^B^*-pool) or are present in free monomers (**C** *^P^*-pool). In each pool, the KaiC monomers exist in four phosphorylation states U - unphosphorylated, T- threonine phosphorylated, S - serine phosphorylated and D - double phosphorylated. In this picture, the concentration of the four phospho-forms of KaiC monomers constitutes a phosphorylation state vector, **C**, with elements 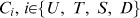. The three pools are defined in the following
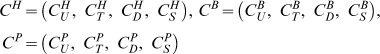
The dynamics of these KaiC monomers are described in equations 1–[Disp-formula pcbi.1002966.e004] 3

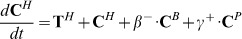
(1)

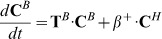
(2)

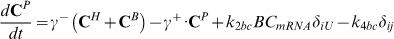
(3)with
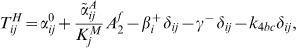
(a)

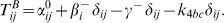
(b)Here, the production of new KaiC molecules occurs within the monomer pool with the rate *k*_2*bc*_ (Eq. 3). For simplicity, we assume that all phospho-forms KaiC of the **C** *^H^*-, **C** *^B^*- and **C** *^P^*-pool are degraded with the same constant rate (*k*_4*bc*_). Further, we disregarded KaiB translation because KaiB has only little effect on dephosphorylation at 30°C [7], [46], [58].

The elements *T_ij_* of transition matrices 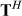 of the hexamer pool and 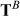 of the KaiBC complexes contain the net transition rates from the KaiC phosphorylation state *j* to *i*, with 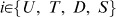. Further, 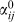 represents the basal phospho-transition rates of KaiC and 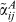 the KaiA-dependent phospho-transition rates of KaiC. The total concentration of the three pools is described by 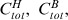 and 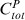. The remaining transition rates are given by
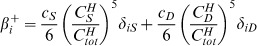
(c)

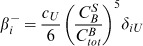
(d)

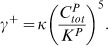
(e)Here, *β^+^* and *β^−^* are the binding rates and dissociation rates of KaiB oligomers and KaiC hexamers, respectively. Assembly of monomers to hexamers increases the concentration of 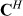 with rate 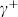. Inversely, KaiC hexamers and KaiBC complexes decompose linearly into the **C** *^P^*-pool with rate 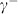. The exchange of KaiC monomers among the hexamers synchronizes the phosphorylation status within the population of KaiC molecules. The Kronecker delta is denoted by *δ_ij_* and the transition rates between the *C_i_* elements with 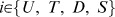 by *c_U_*, *c_S_* and *c_D_*. The hexamer assembly is dependent on the probability that five other monomers of 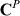 have aggregated to the monomer and is characterized by the Michaelis-Menten constant *K^P^* as well. Moreover, free KaiA are constantly sequestrated through KaiAC complexes.
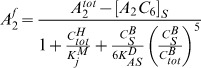
(f)

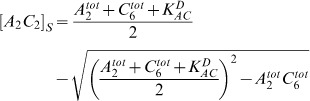
(g)Here, the dissociation constants 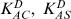 and 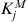 determine the amount of *A_2_C_6_* complexes and of free KaiA dimers 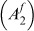. The total amount of KaiA dimers and KaiC hexamers are denoted by 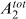 and 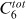, respectively. In the late phosphorylation phase, KaiBC complexes 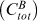 rapidly start to build up. KaiBC complexes with exclusively serine phosphorylated KaiC 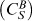 inactivate KaiA. This KaiA sequestration induces the dephosphorylation phase of the system.

In this study, we focus on the TTFL portion of the model. Transcription and translation of the *kai* genes (*kaiA*, *kaiB*, *kaiC*) is based on the Goodwin model [59]. The equations 4–[Disp-formula pcbi.1002966.e033] 6 describe the dynamics of the mRNAs of *kaiA* and *kaiBC* as well as the protein KaiA
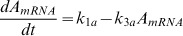
(4)

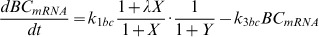
(5)with 


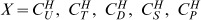
 and 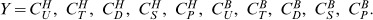


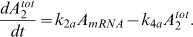
(6)For ease of reading, we changed the nomenclature for the *X* and *Y* in equation (5) into 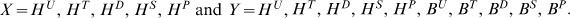


The *kaiA* mRNA does not show any significant circadian rhythm the transcript is therefore synthesized with a constant rate, *k*_1*a*_ (Eq. 4). Transcription of *kaiB* and *kaiC* is lumped into one equation because both genes share the same promoter (Eq. 5). Previous studies assigned KaiC a main role both in suppression and activation of *kaiBC* transcription. In our approach, we use the term for transcriptional activation and transcriptional repression, respectively, showcased in Tab. 1 from Bintu et al. to describe transcription of the *kaiBC* operon [57]; see also Text S1. In particular, we follow the assumption that within the KaiC hexamer pool (**C** *^H^*) one of the phospho-states of KaiC (*X*) turns *kaiBC* transcription on. We additionally assume that one of the phospho-states of KaiC within the hexamer pool or KaiBC complex pool (*Y*) turns it down. The fold-change *λ* is given by the ratio of gene expression (here transcription rate) in the presence and absence of transcription factors. Unknown mechanisms, which regulate transcription of *kaiBC*, are lumped into *λ*. This parameter thus characterizes the effective interactions between the molecular players (Text S1). Moreover, the protein synthesis (constant rate *k*_2_) is dependent on the corresponding synthesized mRNA amount (Eqs. 3, 6). Degradation of mRNAs (*k*_3_) and Kai proteins (*k*_4_) is a reaction of first order as well.

The model was designed as a system of 15 ODEs and implemented using Matlab (R2011b, Mathworks, Cambridge, UK), with a solver for stiff systems (ode15s). We tested different combinations of the phospho-states of KaiC as positive and negative regulators of *kaiBC* transcription. The parameters for the PTO portion were derived from our previous study [16]. Parameters of the TTFL portion were found by fitting the expression profiles of the variables to published expression values [35], using ASAMIN, a MATLAB wrapper routine to ASA (Adaptive Simulated Annealing; www.ingber.com).

Our method of parameter estimation uses a cost function as described in Text S1. We repeated the parameter search from three different initial conditions. For each tested two-loop feedback model, three parameter sets were determined. An optimal parameter set was chosen from these three by comparing the simulated phase relations between *kaiBC* mRNA, UKaiC and PKaiC protein, oscillation rhythms and period of oscillation to the experimental data derived from our image analysis from Figure 2 from Murayama et al. [35] (see Table S1). The parameters of the optimal sets are given in Table S3.

## Supporting Information

Figures S1 Fits for further five two-loop transcriptional feedback models of Group I, which sufficiently reproduce the experimental observed phase relations between *kaiBC* mRNA, unphosphorylated KaiC (UKaiC) and total phosphorylated KaiC (PKaiC) protein and period of oscillation: (**A**) *H^P+^*-*H^U−^*, (**B**) *H^U+^*-*H^T−^*, (**C**) *H^S+^*-*H^D−^*, (**D**) *H^T+^*-*H^S−^*, (**E**) *H^TD+^*-*H^S−^*. In each panel, time-course accumulation of *kaiBC* mRNA (red solid line), unphosphorylated KaiC (UKaiC, blue solid line), and total phosphorylated KaiC protein (PKaiC, black solid line). The levels UKaiC und PKaiC are ratios to total KaiC. The subjective-day phase is from 0 to 12 hours (LL0-12). The subjective-night phase is from 12 to 24 hours (LL12-24). The average level of *kaiBC* transcription was standardized to 1. The symbols represent data from image analysis (see Methods; Table S1). The parameters are given in Table S3. The abbreviations are explained in Figure 1 in the main text.
(TIF)

Figures S2 Fits for two-loop transcriptional feedback models of Group I, which fail to reproduce the experimental observed phase relations between *kaiBC* mRNA, unphosphorylated KaiC (UKaiC) and total phosphorylated KaiC (PKaiC) protein and period of oscillation (part 1): (**A**) *H^T+^*-*H^U−^*, (**B**) *H^D+^*-*H^U−^*, (**C**) *H^S+^*-*H^U−^*, (**D**) *H^U+^*-*H^P−^*. In each panel, time-course accumulation of *kaiBC* mRNA (red solid line), unphosphorylated KaiC (UKaiC, blue solid line), and total phosphorylated KaiC protein (PKaiC, black solid line). The levels UKaiC und PKaiC are ratios to total KaiC. The subjective-day phase is from 0 to 12 hours (LL0-12). The subjective-night phase is from 12 to 24 hours (LL12-24). The average level of *kaiBC* transcription was standardized to 1. The symbols represent data from image analysis (see Methods; Table S1). The parameters are given in Table S3. The abbreviations are explained in Figure 1 in the main text.
(TIF)

Figures S3 Fits for two-loop transcriptional feedback models of Group I, which fail to reproduce the experimental observed phase relations between *kaiBC* mRNA, unphosphorylated KaiC (UKaiC) and total phosphorylated KaiC (PKaiC) protein and period of oscillation (part 2): (**A**) *H^S+^*-*H^T−^*, (**B**) *H^U+^*-*H^D−^*, (**C**) *H^T+^*-*H^D−^*, (**D**) *H^U+^*-*H^S−^*, (**E**) *H^D+^*-*H^S−^*. In each panel, time-course accumulation of *kaiBC* mRNA (red solid line), unphosphorylated KaiC (UKaiC, blue solid line), and total phosphorylated KaiC protein (PKaiC, black solid line). The levels UKaiC und PKaiC are ratios to total KaiC. The subjective-day phase is from 0 to 12 hours (LL0-12). The subjective-night phase is from 12 to 24 hours (LL12-24). The average level of *kaiBC* transcription was standardized to 1. The symbols represent data from image analysis (see Methods; Table S1). The parameters are given in Table S3. The abbreviations are explained in Figure 1 in the main text.
(TIF)

Figures S4 Fits for further five two-loop transcriptional feedback models of Group II, which sufficiently reproduce the experimental observed phase relations between *kaiBC* mRNA, unphosphorylated KaiC (UKaiC) and total phosphorylated KaiC (PKaiC) protein and period of oscillation: (**A**) *H^T+^*-*B^U−^*, (**B**) *H^D+^*-*B^U−^*, (**C**) *H^P+^*-*B^U−^*, (**D**) *H^U+^*-*B^P−^*, (**E**) *H^U+^*-*B^D−^*. In each panel, time-course accumulation of *kaiBC* mRNA (red solid line), unphosphorylated KaiC (UKaiC, blue solid line), and total phosphorylated KaiC protein (PKaiC, black solid line). The levels UKaiC und PKaiC are ratios to total KaiC. The subjective-day phase is from 0 to 12 hours (LL0-12). The subjective-night phase is from 12 to 24 hours (LL12-24). The average level of *kaiBC* transcription was standardized to 1. The symbols represent data from image analysis (see Methods; Table S1). The parameters are given in Table S3. The abbreviations are explained in Figure 1 in the main text.
(TIF)

Figures S5 Fits for two-loop transcriptional feedback models of Group II, which fail to reproduce the experimental observed phase relations between *kaiBC* mRNA, unphosphorylated KaiC (UKaiC) and total phosphorylated KaiC (PKaiC) protein and period of oscillation (part 1): (**A**) *H^S+^*-*B^U−^*, (**B**) *H^U+^*-*B^T−^*, (**C**) *H^S+^*-*B^T−^*, (**D**) *H^T+^*-*B^D−^*, (**E**) *H^S+^*-*B^D−^*. In each panel, time-course accumulation of *kaiBC* mRNA (red solid line), unphosphorylated KaiC (UKaiC, blue solid line), and total phosphorylated KaiC protein (PKaiC, black solid line). The levels UKaiC und PKaiC are ratios to total KaiC. The subjective-day phase is from 0 to 12 hours (LL0-12). The subjective-night phase is from 12 to 24 hours (LL12-24). The average level of *kaiBC* transcription was standardized to 1. The symbols represent data from image analysis (see Methods; Table S1). The parameters are given in Table S3. The abbreviations are explained in Figure 1 in the main text.
(TIF)

Figures S6 Fits for two-loop transcriptional feedback models of Group II, which fail to reproduce the experimental observed phase relations between *kaiBC* mRNA, unphosphorylated KaiC (UKaiC) and total phosphorylated KaiC (PKaiC) protein and period of oscillation (part 2): (**A**) *H^U+^*-*B^S−^*, (**B**) *H^T+^*-*B^S−^*, (**C**) *H^D+^*-*B^S−^*, (**D**) *H^TD+^*-*B^S−^*. In each panel, time-course accumulation of *kaiBC* mRNA (red solid line), unphosphorylated KaiC (UKaiC, blue solid line), and total phosphorylated KaiC protein (PKaiC, black solid line). The levels UKaiC und PKaiC are ratios to total KaiC. The subjective-day phase is from 0 to 12 hours (LL0-12). The subjective-night phase is from 12 to 24 hours (LL12-24). The average level of *kaiBC* transcription was standardized to 1. The symbols represent data from image analysis (see Methods; Table S1). The parameters are given in Table S3. The abbreviations are explained in Figure 1 in the main text.
(TIF)

Figure S7 Predicted time-series of *kaiBC* expression and KaiC phosphorylation for the models of Group I and II, which show circadian oscillation of *kaiBC* mRNA, UKaiC protein and PKaiC protein levels with consistent peak concentration and phase relation (Figure 1, S1, S4) but fail to recapitulate downregulation of *kaiBC* expression upon *kaiA* inactivation. (**A–C**) Group I models: (**A**) *H^U+^*-*H^T−^*, (**B**) *H^S+^*-*H^D−^*, (**C**) *H^T+^*-*H^S−^*. (**D–H**) Group II models: (**D**) *H^T+^*-*B^U−^*, (**E**) *H^D+^*-*B^U−^*, (**F**) *H^P+^*-*B^U−^*, (**G**) *H^U+^*-*B^P−^*, (**H**) *H^U+^*-*B^D−^*.
(TIF)

Figure S8 Initial dynamics of the transcriptional KaiC feed-back species in simulated *kaiA*-knockout mutants. Each panel depicts the simulated expression dynamics of the positive transcriptional regulator, the negative transcriptional regulator and *kaiBC* mRNA for the first days in LL shortly after *kaiA* transcription was removed from the (A) *H^T+^*-*H^D−^*, (B) *H^TD+^*-*H^S−^* and (C) *H^D+^*-*H^S−^* models.
(TIF)

Figure S9 Effect of depletion and overexpression of the *kaiA* gene on the expression dynamics of *kaiBC* mRNA and KaiC phosphorylation predicted from the *H^D+^*-*B^T−^* model. Deletion of the *kaiA* gene was simulated through setting the *kaiA* transcription rate to zero whereas overexpression was achieved by increasing the rate 100-fold ().
(TIF)

Figures S10 Fits for two-loop transcriptional feedback models of Group II, which fail to reproduce the experimental observed phase relations between *kaiBC* mRNA, unphosphorylated KaiC (UKaiC) and total phosphorylated KaiC (PKaiC) protein and period of oscillation (part 3): (**A**) *H^T+^*-*B^SU−^*, (**B**) *D^T+^*-*B^SU−^*, (**C**) *H^TD+^*-*B^SU−^*. In each panel, time-course accumulation of *kaiBC* mRNA (red solid line), unphosphorylated KaiC (UKaiC, blue solid line), and total phosphorylated KaiC protein (PKaiC, black solid line). The levels UKaiC und PKaiC are ratios to total KaiC. The subjective-day phase is from 0 to 12 hours (LL0-12). The subjective-night phase is from 12 to 24 hours (LL12-24). The average level of *kaiBC* transcription was standardized to 1. The symbols represent data from image analysis (see Methods; Table S1). The parameters are given in Table S3. The abbreviations are explained in Figure 1 in the main text.
(TIF)

Figure S11 Initial dynamics of the transcriptional KaiC feed-back species in simulated KaiC overexpression mutants. KaiC was simulated through increasing the translational rate of unphosphorylated KaiC monomers at time of minimal *kaiBC* expression. Each panel depicts the simulated expression dynamics of the positive transcriptional regulator, the negative transcriptional regulator and *kaiBC* mRNA for the first days in LL shortly after *KaiC* overexpression was induced in the (A) *H^U+^*-*H^T−^* and (B) *H^S+^*-*H^D−^* models.
(TIF)

Table S1 Data from the image analysis.
(DOC)

Table S2 Values of the simulated peak phases and period for the tested two-loop feedback model. For each model, the values base upon the optimal parameter set chosen (see Methods). The models highlighted in grey were analyzed further.
(DOC)

Table S3 List of the optimal parameter values of the TTFL.
(DOC)

Text S1 Supporting Information. More detailed information on choice of the activation and repression term in equation (5), cost function, binomial distribution calculation and simulations of *kai* mutants.
(DOC)
