# Supplementary figures and images for: Revealing a Two-Loop Transcriptional Feedback Mechanism in the Cyanobacterial Circadian Clock
Source: PLoS Comput Biol. 2013 Mar 14;9(3):e1002966. doi: 10.1371/journal.pcbi.1002966 (PMC3597532; doi:10.1371/journal.pcbi.1002966)

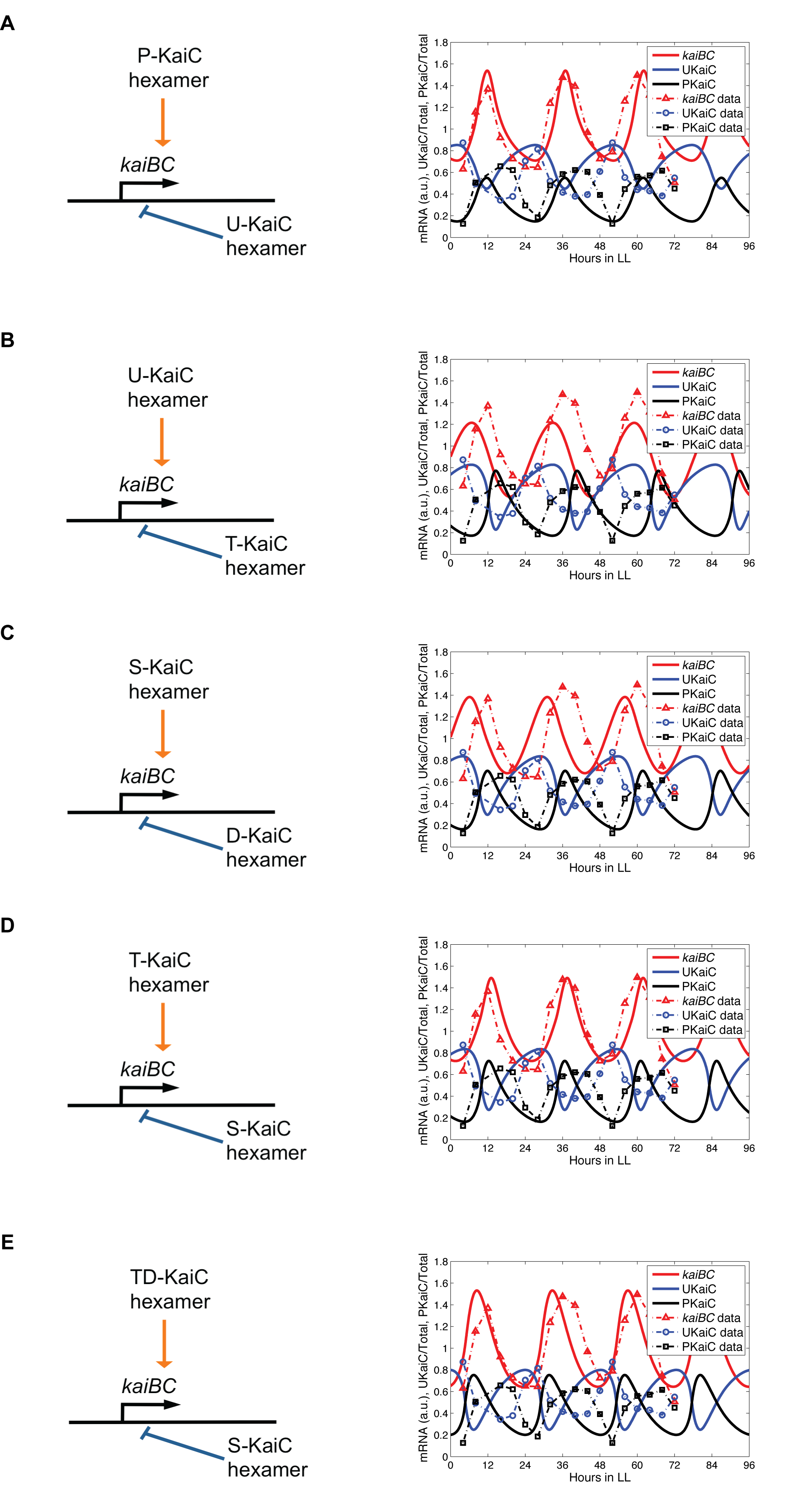

Supplement: Figures S1 — Fits for further five two-loop transcriptional feedback models of Group I, which sufficiently reproduce the experimental observed phase relations between kaiBC mRNA, unphosphorylated KaiC (UKaiC) and total phosphorylated KaiC (PKaiC) protein and period of oscillation: (A) HP+-HU−, (B) HU+-HT−, (C) HS+-HD−, (D) HT+-HS−, (E) HTD+-HS−. In each panel, time-course accumulation of kaiBC mRNA (red solid line), unphosphorylated KaiC (UKaiC, blue solid line), and total phosphorylated KaiC protein (PKaiC, black solid line). The levels UKaiC und PKaiC are ratios to total KaiC. The subjective-day phase is from 0 to 12 hours (LL0-12). The subjective-night phase is from 12 to 24 hours (LL12-24). The average level of kaiBC transcription was standardized to 1. The symbols represent data from image analysis (see Methods; Table S1). The parameters are given in Table S3. The abbreviations are explained in Figure 1 in the main text. (TIF) [file pcbi.1002966.s001.tif]

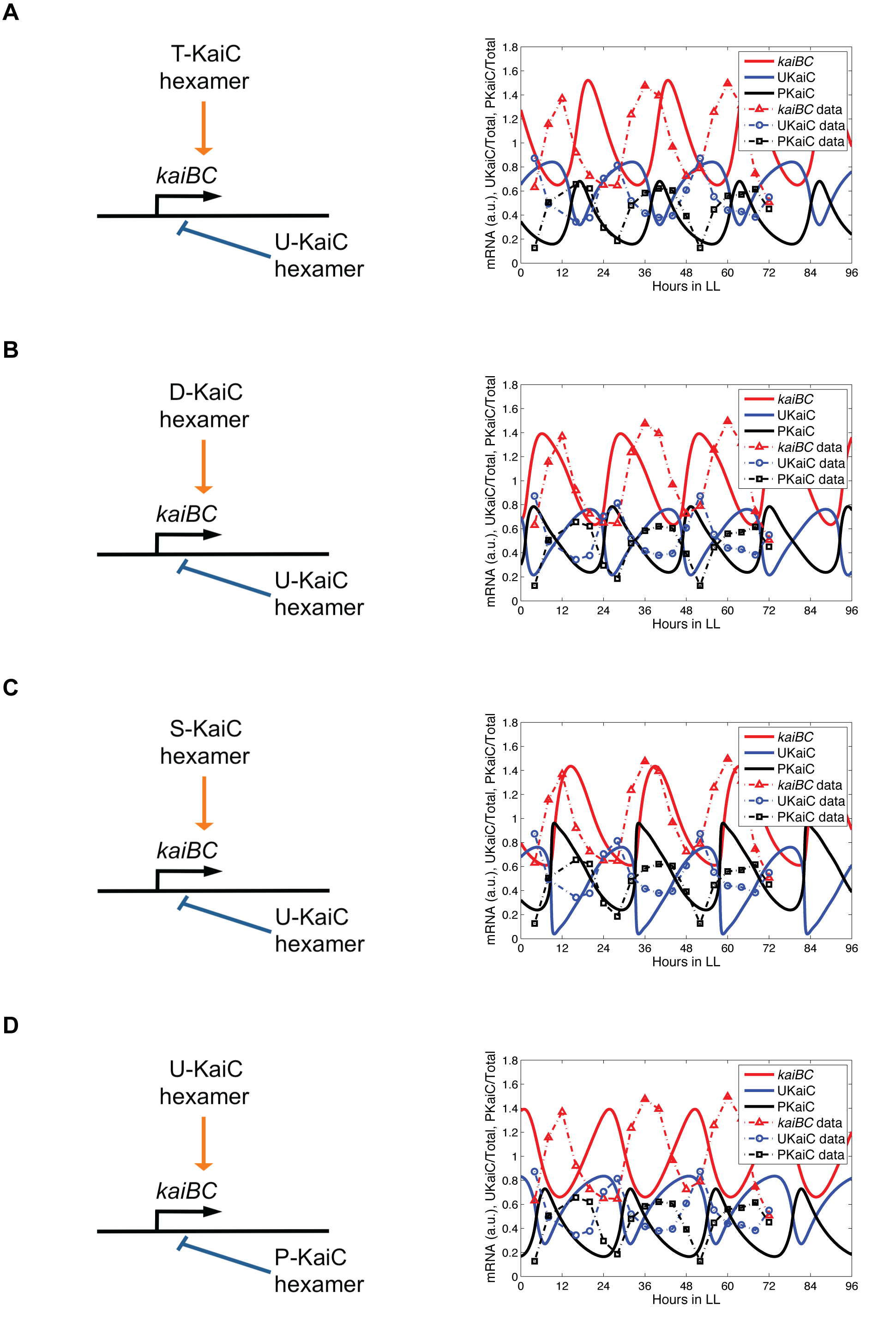

Supplement: Figures S2 — Fits for two-loop transcriptional feedback models of Group I, which fail to reproduce the experimental observed phase relations between kaiBC mRNA, unphosphorylated KaiC (UKaiC) and total phosphorylated KaiC (PKaiC) protein and period of oscillation (part 1): (A) HT+-HU−, (B) HD+-HU−, (C) HS+-HU−, (D) HU+-HP−. In each panel, time-course accumulation of kaiBC mRNA (red solid line), unphosphorylated KaiC (UKaiC, blue solid line), and total phosphorylated KaiC protein (PKaiC, black solid line). The levels UKaiC und PKaiC are ratios to total KaiC. The subjective-day phase is from 0 to 12 hours (LL0-12). The subjective-night phase is from 12 to 24 hours (LL12-24). The average level of kaiBC transcription was standardized to 1. The symbols represent data from image analysis (see Methods; Table S1). The parameters are given in Table S3. The abbreviations are explained in Figure 1 in the main text. (TIF) [file pcbi.1002966.s002.tif]

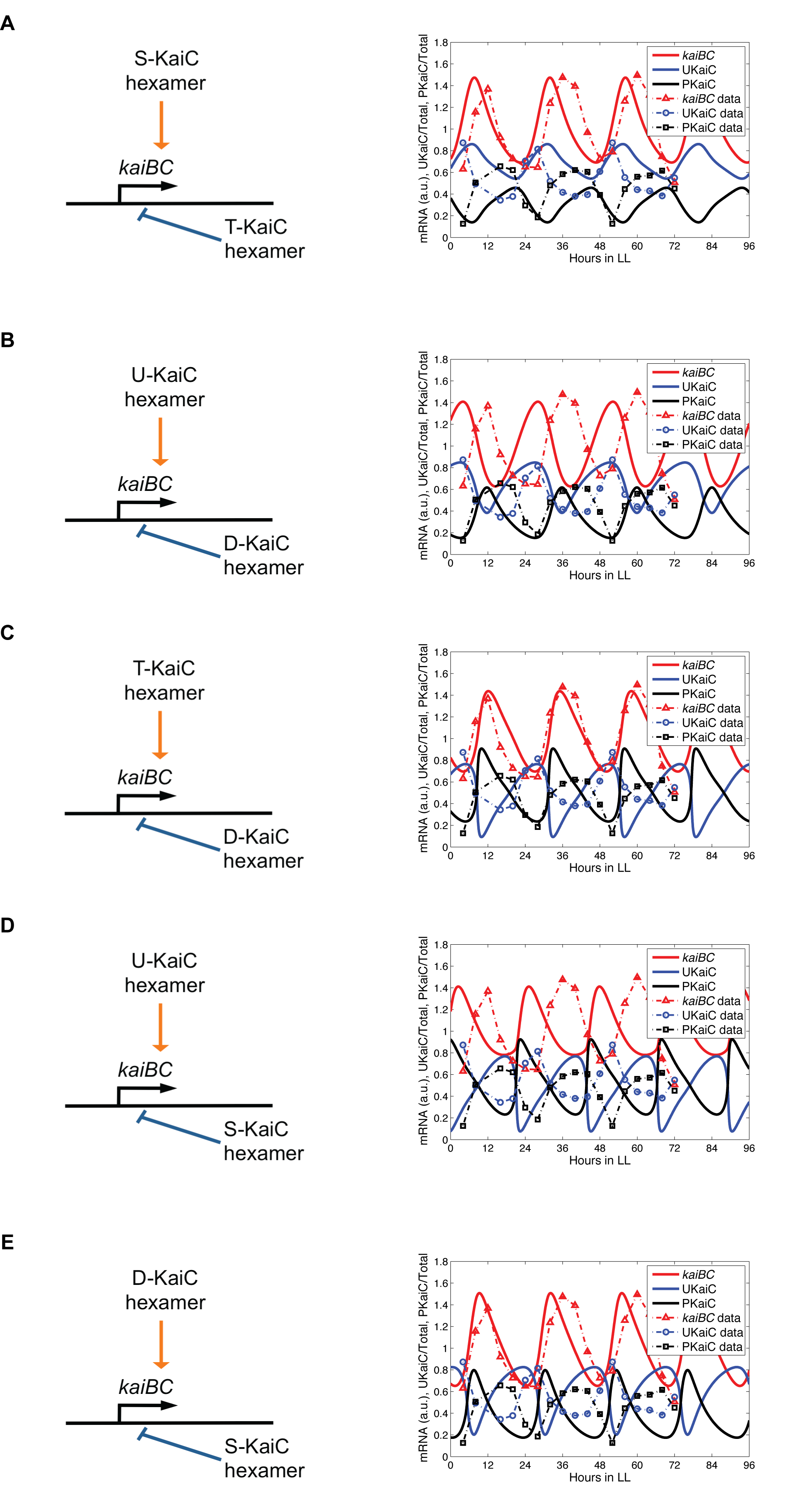

Supplement: Figures S3 — Fits for two-loop transcriptional feedback models of Group I, which fail to reproduce the experimental observed phase relations between kaiBC mRNA, unphosphorylated KaiC (UKaiC) and total phosphorylated KaiC (PKaiC) protein and period of oscillation (part 2): (A) HS+-HT−, (B) HU+-HD−, (C) HT+-HD−, (D) HU+-HS−, (E) HD+-HS−. In each panel, time-course accumulation of kaiBC mRNA (red solid line), unphosphorylated KaiC (UKaiC, blue solid line), and total phosphorylated KaiC protein (PKaiC, black solid line). The levels UKaiC und PKaiC are ratios to total KaiC. The subjective-day phase is from 0 to 12 hours (LL0-12). The subjective-night phase is from 12 to 24 hours (LL12-24). The average level of kaiBC transcription was standardized to 1. The symbols represent data from image analysis (see Methods; Table S1). The parameters are given in Table S3. The abbreviations are explained in Figure 1 in the main text. (TIF) [file pcbi.1002966.s003.tif]

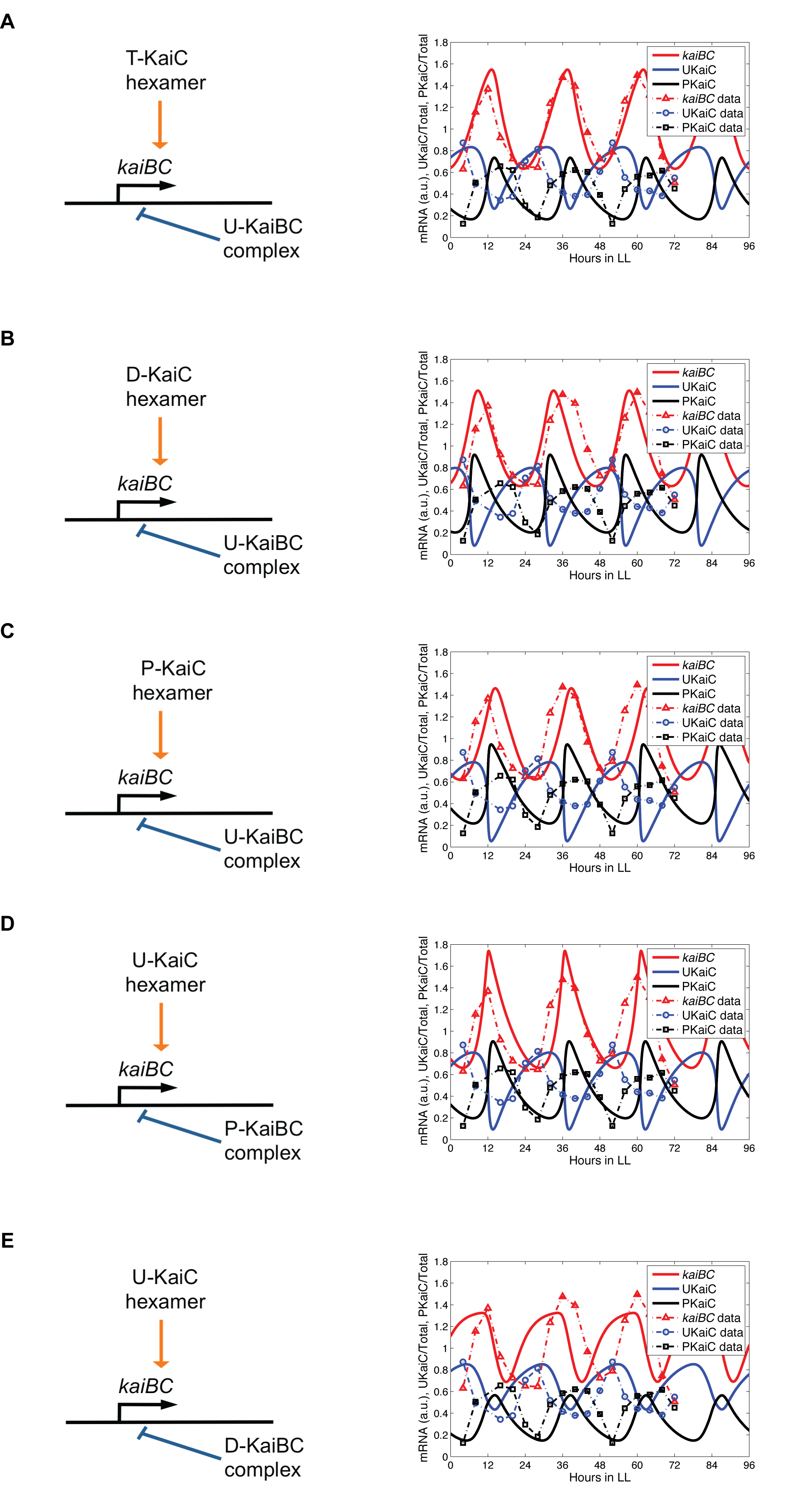

Supplement: Figures S4 — Fits for further five two-loop transcriptional feedback models of Group II, which sufficiently reproduce the experimental observed phase relations between kaiBC mRNA, unphosphorylated KaiC (UKaiC) and total phosphorylated KaiC (PKaiC) protein and period of oscillation: (A) HT+-BU−, (B) HD+-BU−, (C) HP+-BU−, (D) HU+-BP−, (E) HU+-BD−. In each panel, time-course accumulation of kaiBC mRNA (red solid line), unphosphorylated KaiC (UKaiC, blue solid line), and total phosphorylated KaiC protein (PKaiC, black solid line). The levels UKaiC und PKaiC are ratios to total KaiC. The subjective-day phase is from 0 to 12 hours (LL0-12). The subjective-night phase is from 12 to 24 hours (LL12-24). The average level of kaiBC transcription was standardized to 1. The symbols represent data from image analysis (see Methods; Table S1). The parameters are given in Table S3. The abbreviations are explained in Figure 1 in the main text. (TIF) [file pcbi.1002966.s004.tif]

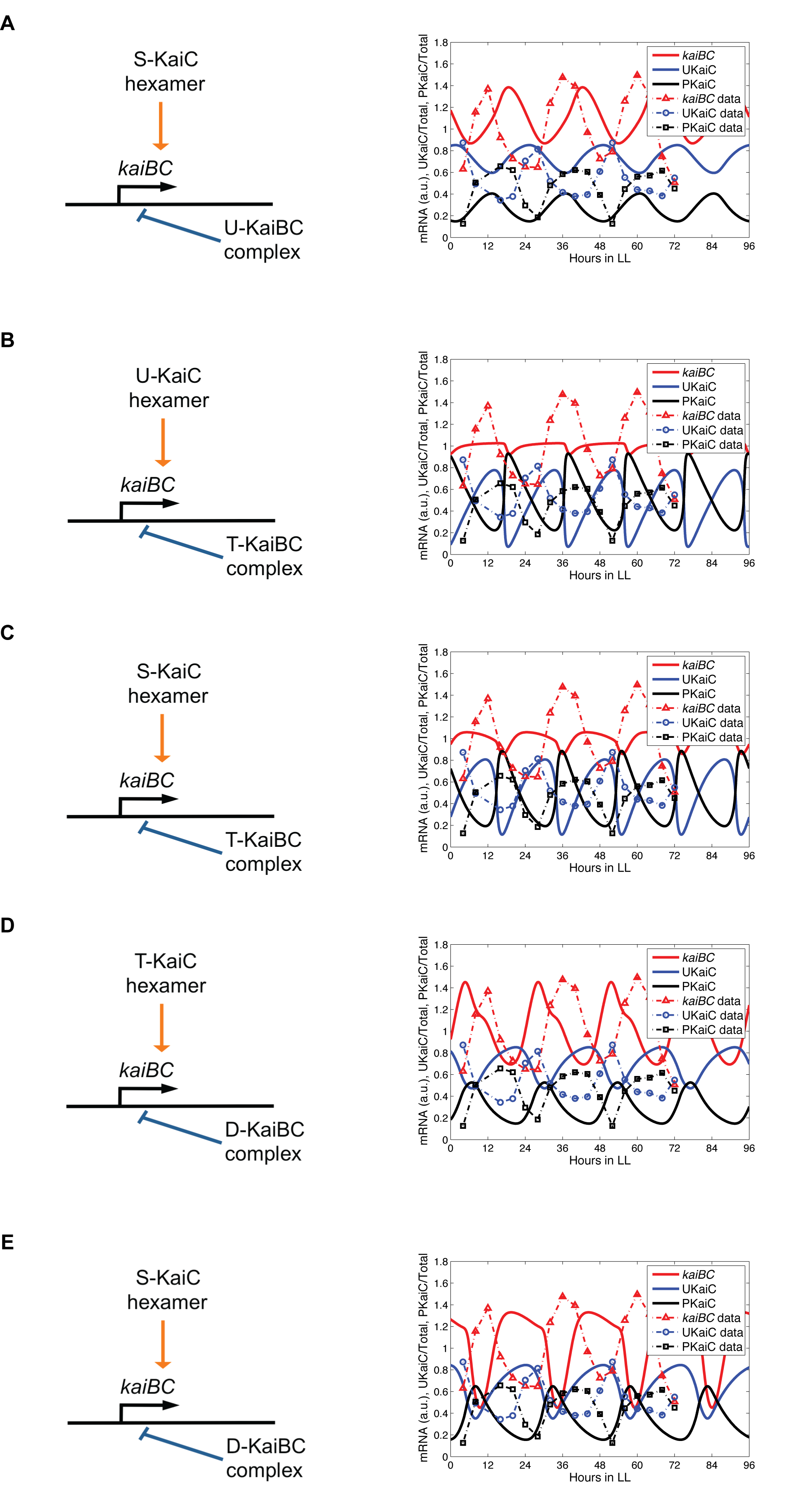

Supplement: Figures S5 — Fits for two-loop transcriptional feedback models of Group II, which fail to reproduce the experimental observed phase relations between kaiBC mRNA, unphosphorylated KaiC (UKaiC) and total phosphorylated KaiC (PKaiC) protein and period of oscillation (part 1): (A) HS+-BU−, (B) HU+-BT−, (C) HS+-BT−, (D) HT+-BD−, (E) HS+-BD−. In each panel, time-course accumulation of kaiBC mRNA (red solid line), unphosphorylated KaiC (UKaiC, blue solid line), and total phosphorylated KaiC protein (PKaiC, black solid line). The levels UKaiC und PKaiC are ratios to total KaiC. The subjective-day phase is from 0 to 12 hours (LL0-12). The subjective-night phase is from 12 to 24 hours (LL12-24). The average level of kaiBC transcription was standardized to 1. The symbols represent data from image analysis (see Methods; Table S1). The parameters are given in Table S3. The abbreviations are explained in Figure 1 in the main text. (TIF) [file pcbi.1002966.s005.tif]

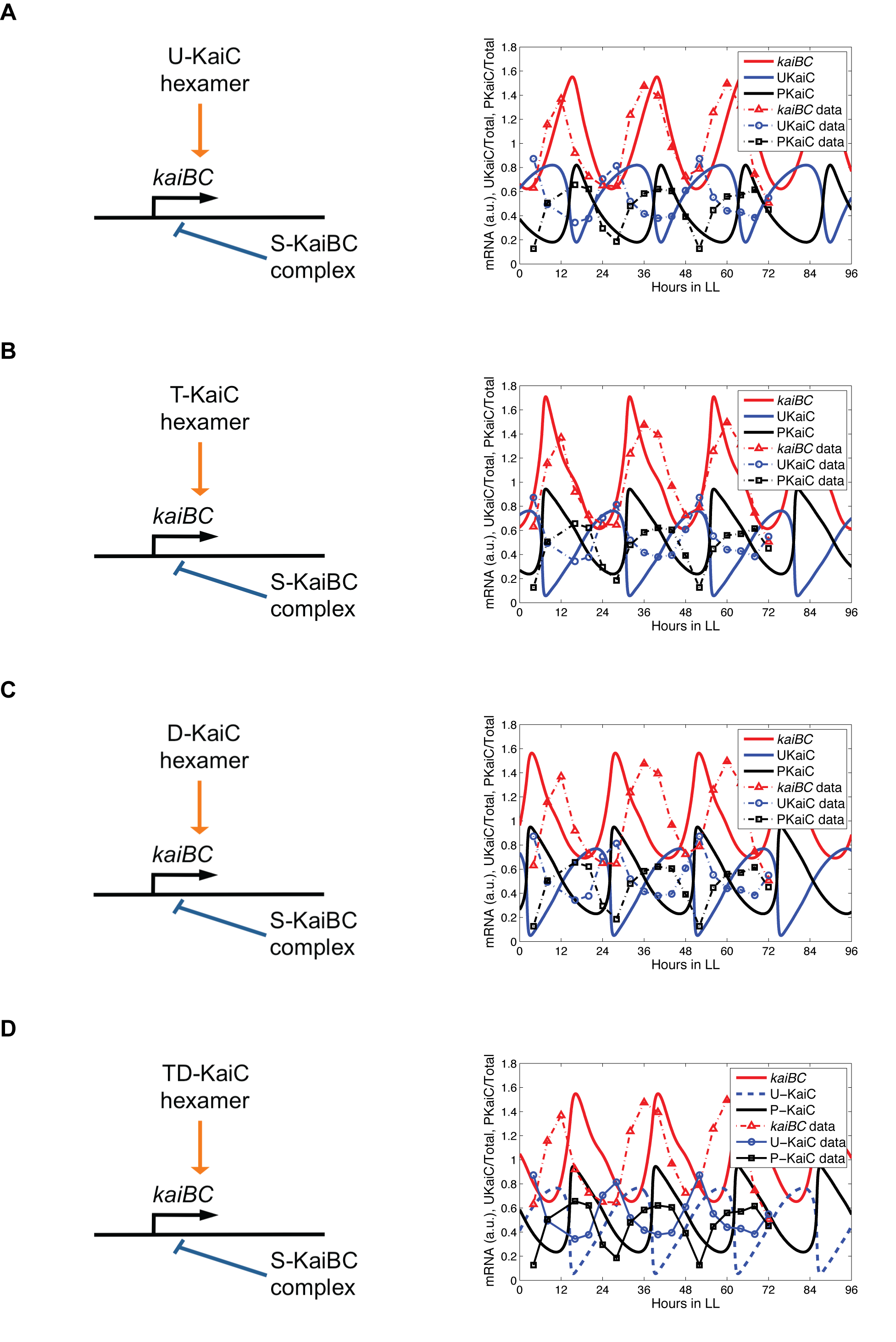

Supplement: Figures S6 — Fits for two-loop transcriptional feedback models of Group II, which fail to reproduce the experimental observed phase relations between kaiBC mRNA, unphosphorylated KaiC (UKaiC) and total phosphorylated KaiC (PKaiC) protein and period of oscillation (part 2): (A) HU+-BS−, (B) HT+-BS−, (C) HD+-BS−, (D) HTD+-BS−. In each panel, time-course accumulation of kaiBC mRNA (red solid line), unphosphorylated KaiC (UKaiC, blue solid line), and total phosphorylated KaiC protein (PKaiC, black solid line). The levels UKaiC und PKaiC are ratios to total KaiC. The subjective-day phase is from 0 to 12 hours (LL0-12). The subjective-night phase is from 12 to 24 hours (LL12-24). The average level of kaiBC transcription was standardized to 1. The symbols represent data from image analysis (see Methods; Table S1). The parameters are given in Table S3. The abbreviations are explained in Figure 1 in the main text. (TIF) [file pcbi.1002966.s006.tif]

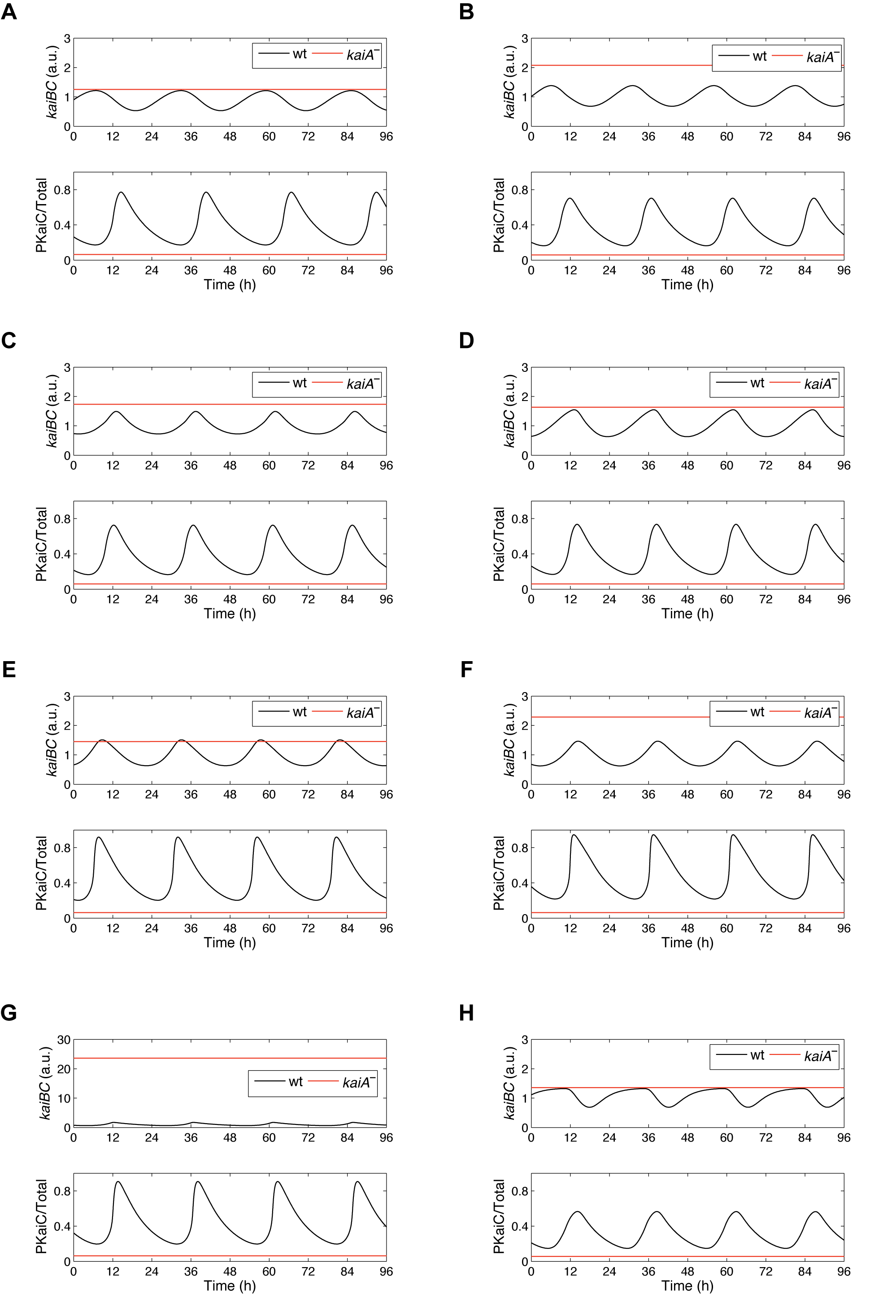

Supplement: Figure S7 — Predicted time-series of kaiBC expression and KaiC phosphorylation for the models of Group I and II, which show circadian oscillation of kaiBC mRNA, UKaiC protein and PKaiC protein levels with consistent peak concentration and phase relation (Figure 1, S1, S4) but fail to recapitulate downregulation of kaiBC expression upon kaiA inactivation. (A–C) Group I models: (A) HU+-HT−, (B) HS+-HD−, (C) HT+-HS−. (D–H) Group II models: (D) HT+-BU−, (E) HD+-BU−, (F) HP+-BU−, (G) HU+-BP−, (H) HU+-BD−. (TIF) [file pcbi.1002966.s007.tif]

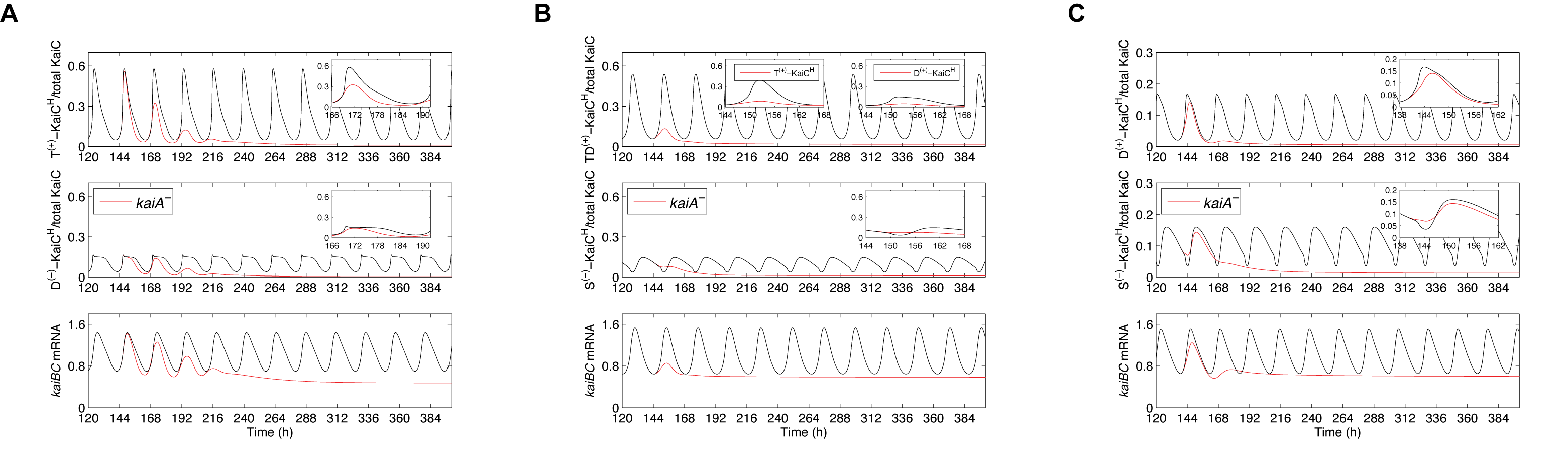

Supplement: Figure S8 — Initial dynamics of the transcriptional KaiC feed-back species in simulated kaiA-knockout mutants. Each panel depicts the simulated expression dynamics of the positive transcriptional regulator, the negative transcriptional regulator and kaiBC mRNA for the first days in LL shortly after kaiA transcription was removed from the (A) HT+-HD−, (B) HTD+-HS− and (C) HD+-HS− models. (TIF) [file pcbi.1002966.s008.tif]

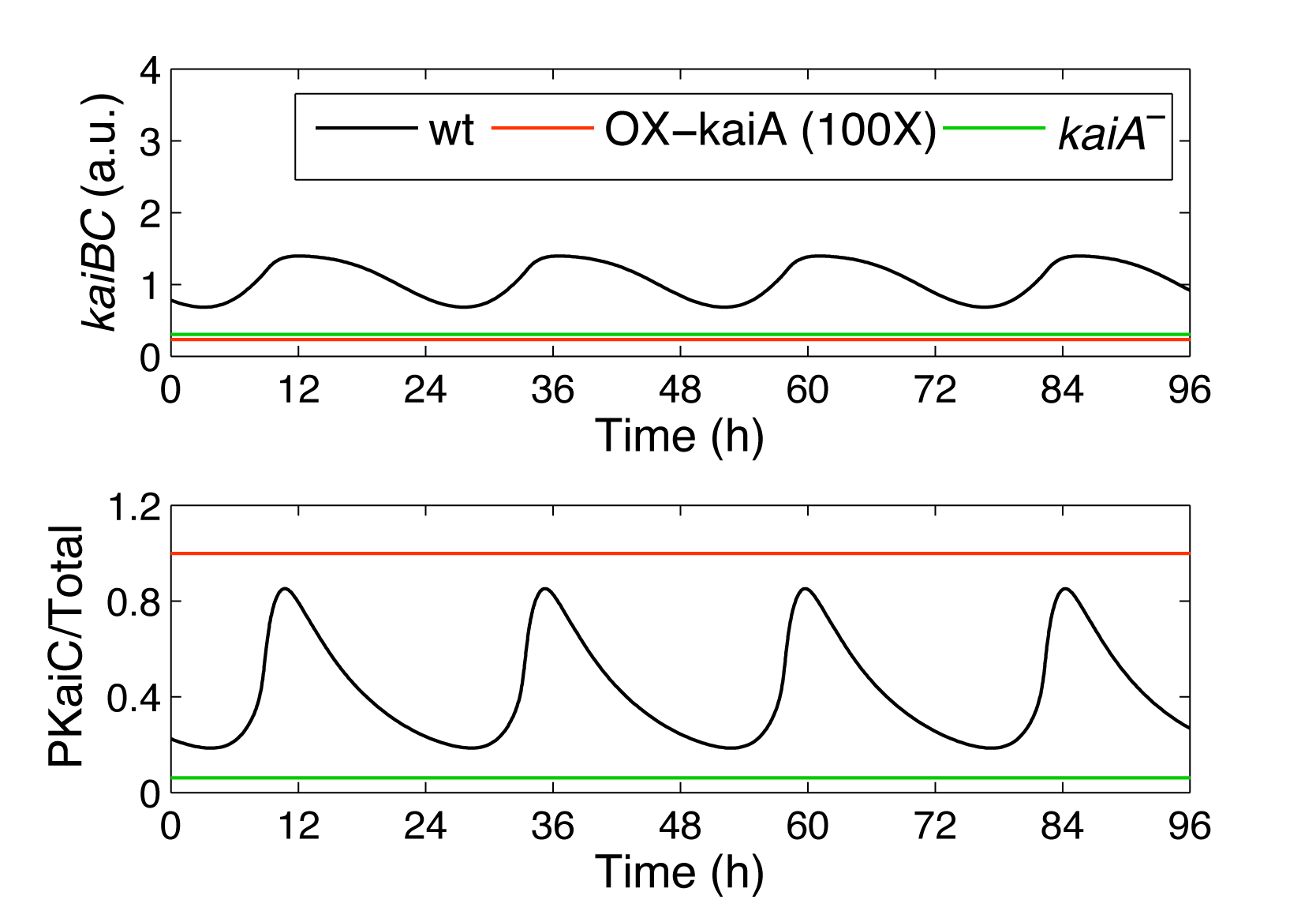

Supplement: Figure S9 — Effect of depletion and overexpression of the kaiA gene on the expression dynamics of kaiBC mRNA and KaiC phosphorylation predicted from the HD+-BT− model. Deletion of the kaiA gene was simulated through setting the kaiA transcription rate to zero whereas overexpression was achieved by increasing the rate 100-fold (). (TIF) [file pcbi.1002966.s009.tif]

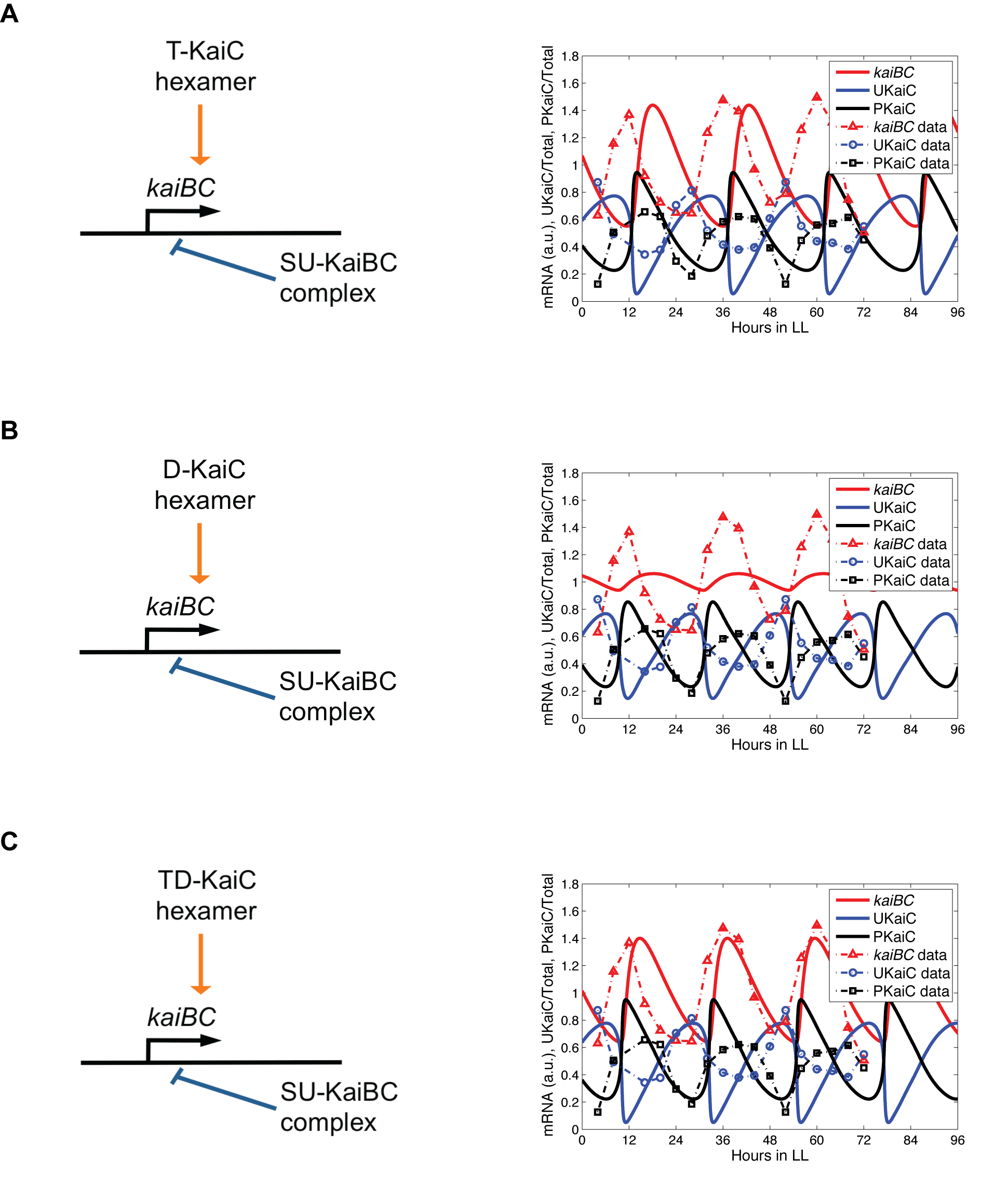

Supplement: Figures S10 — Fits for two-loop transcriptional feedback models of Group II, which fail to reproduce the experimental observed phase relations between kaiBC mRNA, unphosphorylated KaiC (UKaiC) and total phosphorylated KaiC (PKaiC) protein and period of oscillation (part 3): (A) HT+-BSU−, (B) DT+-BSU−, (C) HTD+-BSU−. In each panel, time-course accumulation of kaiBC mRNA (red solid line), unphosphorylated KaiC (UKaiC, blue solid line), and total phosphorylated KaiC protein (PKaiC, black solid line). The levels UKaiC und PKaiC are ratios to total KaiC. The subjective-day phase is from 0 to 12 hours (LL0-12). The subjective-night phase is from 12 to 24 hours (LL12-24). The average level of kaiBC transcription was standardized to 1. The symbols represent data from image analysis (see Methods; Table S1). The parameters are given in Table S3. The abbreviations are explained in Figure 1 in the main text. (TIF) [file pcbi.1002966.s010.tif]

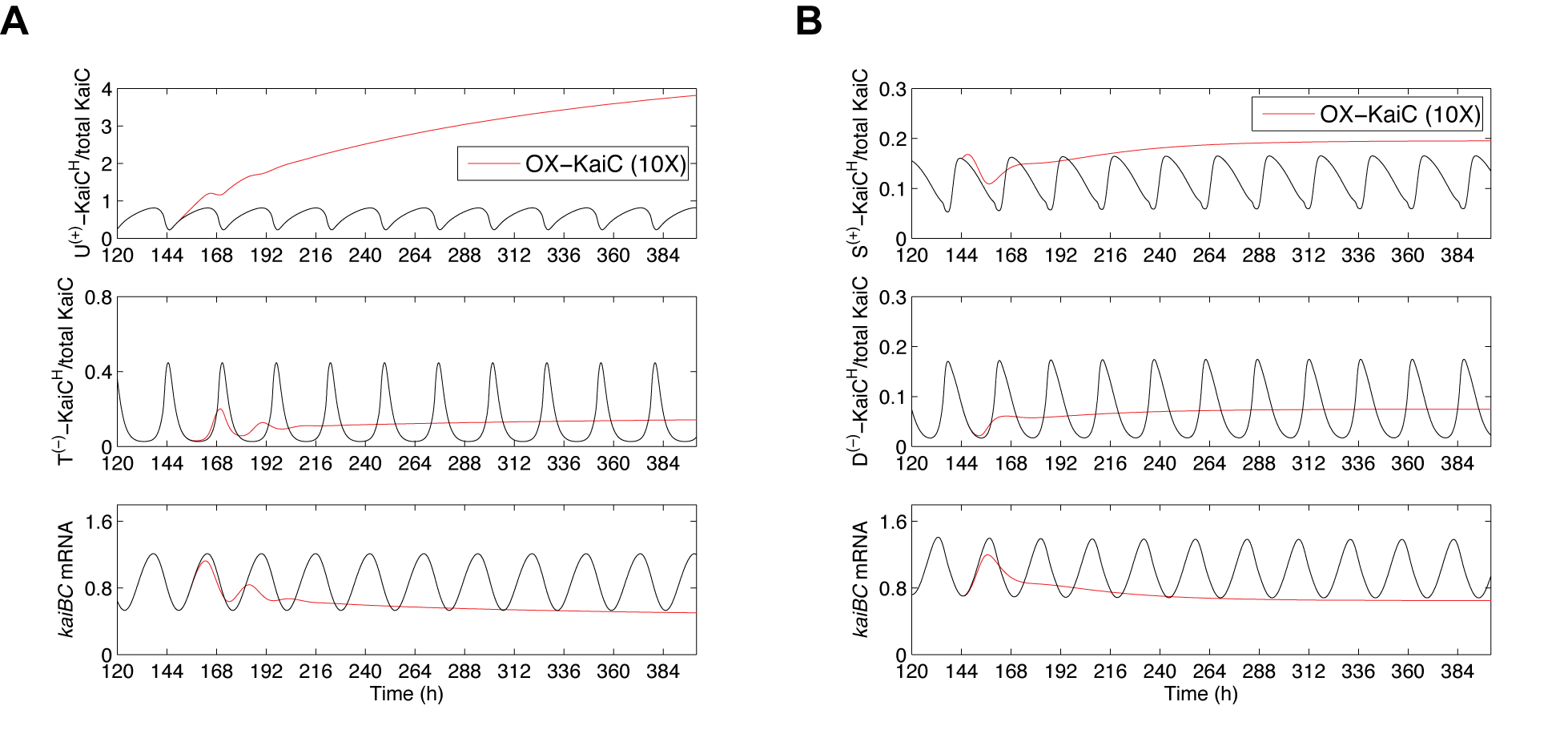

Supplement: Figure S11 — Initial dynamics of the transcriptional KaiC feed-back species in simulated KaiC overexpression mutants. KaiC was simulated through increasing the translational rate of unphosphorylated KaiC monomers at time of minimal kaiBC expression. Each panel depicts the simulated expression dynamics of the positive transcriptional regulator, the negative transcriptional regulator and kaiBC mRNA for the first days in LL shortly after KaiC overexpression was induced in the (A) HU+-HT− and (B) HS+-HD− models. (TIF) [file pcbi.1002966.s011.tif]
