# Supplementary material for: Revealing a Two-Loop Transcriptional Feedback Mechanism in the Cyanobacterial Circadian Clock
Source: PLoS Comput Biol. 2013 Mar 14;9(3):e1002966. doi: 10.1371/journal.pcbi.1002966 (PMC3597532; doi:10.1371/journal.pcbi.1002966)
Supplement: Table S1 — Data from the image analysis. (DOC) [file pcbi.1002966.s012.doc]

**Table S1.** Data from the image analysis.

| ***t_n_* (h)** | ***kaiBC* mRNA** | **Unphosphorylated KaiC/total KaiC** | **Total phosphorylated KaiC/total KaiC** |
| --- | --- | --- | --- |
| 04 | 0.62125162 | 0.87374092 | 0.12625907 |
| 08 | 1.15677636 | 0.49368264 | 0.50631735 |
| 12 | 1.36881316 | - | - |
| 16 | 0.91966830 | 0.34337349 | 0.65662650 |
| 20 | 0.72473438 | 0.37832268 | 0.62167731 |
| 24 | 0.65010365 | 0.70483948 | 0.29516051 |
| 28 | 0.64602228 | 0.81459181 | 0.18540818 |
| 32 | 1.23568281 | 0.51944883 | 0.48055117 |
| 36 | 1.47512308 | 0.41605039 | 0.58394960 |
| 40 | 1.39349572 | 0.37878787 | 0.62121212 |
| 44 | 0.96767297 | 0.39486552 | 0.60513447 |
| 48 | 0.72473438 | 0.60819197 | 0.39180802 |
| 52 | 0.78906452 | 0.87351895 | 0.12648104 |
| 56 | 1.25686706 | 0.55324838 | 0.44675161 |
| 60 | 1.49514122 | 0.44064274 | 0.55935725 |
| 64 | 1.31264576 | 0.42839371 | 0.57160628 |
| 68 | 0.74572428 | 0.38442565 | 0.61557434 |
| 72 | 0.50647836 | 0.54872597 | 0.45127402 |
