# Supplementary material for: Revealing a Two-Loop Transcriptional Feedback Mechanism in the Cyanobacterial Circadian Clock
Source: PLoS Comput Biol. 2013 Mar 14;9(3):e1002966. doi: 10.1371/journal.pcbi.1002966 (PMC3597532; doi:10.1371/journal.pcbi.1002966)
Supplement: Table S2 — Values of the simulated peak phases and period for the tested two-loop feedback model. For each model, the values base upon the optimal parameter set chosen (see Methods). The models highlighted in grey were analyzed further. (DOC) [file pcbi.1002966.s013.doc]

**Table S2.** **Values of the simulated peak phases and period for the tested two-loop feedback model.** For each model, the values base on the optimal parameter set chosen
(see Methods).

|  | **Peak phases in LL (h)** | | |  |
| --- | --- | --- | --- | --- |
| **Group I Models** | ***kaiBC* mRNA** | **UKaiC** | **PKaiC** | **Period (h)** |
| *H^U+^*-*H^P-^* | 0.9 | 24.4 | 7.0 | 24.8 |
| *H^T+^*-*H^U-^* | 19.5 | 8.0 | 17.2 | 23.3 |
| *H^D+^*-*H^U-^* | 6.1 | 18.5 | 4.0 | 22.3 |
| *H^S+^*-*H^U-^* | 14.5 | 4.2 | 9.7 | 24.5 |
| *H^TD+^*-*H^U-^* | 13.0 | 2.2 | 10.8 | 24.4 |
| *H^P+^*-*H^U-^* | 11.8 | 1.4 | 11.8 | 25.2 |
| *H^U+^*-*H^T-^* | 6.7 | 6.7 | 14.5 | 26.1 |
| *H^D+^*-*H^T-^* | 17.7 | 18.0 | 1.5 | 24.6 |
| *H^S+^*-*H^T-^* | 7.7 | 8.4 | 19.6 | 24.3 |
| *H^U+^*-*H^D-^* | 4.0 | 3.8 | 11.6 | 24.1 |
| *H^T+^*-*H^D-^* | 12.2 | 3.6 | 10.1 | 23.0 |
| *H^S+^*-*H^D-^* | 6.1 | 4.3 | 11.7 | 24.9 |
| *H^U+^*-*H^S-^* | 2.4 | 17.0 | 22.5 | 22.7 |
| *H^T+^*-*H^S-^* | 13.0 | 4.0 | 12.3 | 24.4 |
| *H^D+^*-*H^S-^* | 9.3 | 22.5 | 7.5 | 22.9 |
| *H^TD+^*-*H^S-^* | 8.4 | 0.0 | 7.6 | 24.3 |
| *H^TD+^*-*H^SU-^* | 14.8 | 5.4 | 11.2 | 22.4 |
| *H^T+^*-*H^SU-^* | 18.0 | 8.1 | 14.0 | 24.6 |
| *H^D+^*-*H^SU-^* | 18.3 | 5.9 | 11.7 | 21.7 |
| **Group II Models** |  | | | |
| *H^U+^*-*B^P-^* | 12.2 | 7.0 | 13.6 | 24.5 |
| *H^T+^*-*B^U-^* | 13.2 | 6.6 | 14.1 | 24.4 |
| *H^D+^*-*B^U-^* | 8.8 | 1.2 | 7.7 | 24.4 |
| *H^S+^*-*B^U-^* | 18.6 | 1.3 | 13.2 | 23.7 |
| *H^TD+^*-*B^U-^* | 15.1 | 12.1 | 17.6 | 24.4 |
| *H^P+^*-*B^U-^* | 14.3 | 7.1 | 13.1 | 24.5 |
| *H^U+^*-*B^T-^* | 15.1 | 14.2 | 18.5 | 19.3 |
| *H^D+^*-*B^T-^* | 12.1 | 3.5 | 10.7 | 24.6 |
| *H^S+^*-*B^T-^* | 5.3 | 11.3 | 16.5 | 19.2 |
| *H^U+^*-*B^D-^* | 10.1 | 5.4 | 14.2 | 24.4 |
| *H^T+^*-*B^D-^* | 4.8 | 19.4 | 7.0 | 23.5 |
| *H^S+^*-*B^D-^* | 19.5 | 0.0 | 8.0 | 24.9 |
| *H^U+^*-*B^S-^* | 15.2 | 9.4 | 16.5 | 24.4 |
| *H^T+^*-*B^S-^* | 7.5 | 2.2 | 7.8 | 24.3 |
| *H^D+^*-*B^S-^* | 3.5 | 21.7 | 3.2 | 24.2 |
| *H^TD+^*-*B^S-^* | 16.2 | 9.9 | 15.7 | 23.8 |
