# Supplementary material for: Revealing a Two-Loop Transcriptional Feedback Mechanism in the Cyanobacterial Circadian Clock
Source: PLoS Comput Biol. 2013 Mar 14;9(3):e1002966. doi: 10.1371/journal.pcbi.1002966 (PMC3597532; doi:10.1371/journal.pcbi.1002966)
Supplement: Table S3 — List of the optimal parameter values of the TTFL. (DOC) [file pcbi.1002966.s014.doc]

**Table S3.** **List of the optimal parameter values of the TTFL.**

| **Model Group I** (see also Methods; Table 1) | ***k*_1_*_a_***, *kaiA* transcription rate | ***k*_1_*_bc_***, *kaiBC* transcription rate | ***k*_2_*_a_***, *kaiA* translation rate | ***k*_2_*_bc_***, *kaiBC* translation rate | ***k*_3_*_a_***, *kaiA* degradation rate | ***k*_3_*_bc_***, *kaiBC* degradation rate | ***k*_4_*_a_***, KaiA degradation rate | ***k*_4_*_bc_***, KaiC degradation rate | ***λ***, Fold-change |
| --- | --- | --- | --- | --- | --- | --- | --- | --- | --- |
| *H^U+^*-*H^P-^* | 0.8198 | 0.6810 | 2.3674 | 0.6654 | 0.2776 | 0.2098 | 0.6193 | 0.0123 | 4.6102 |
| *H^T+^*-*H^U-^* | 0.1551 | 0.9213 | 0.5206 | 0.3543 | 0.1023 | 0.2168 | 0.3082 | 0.0251 | 3.0267 |
| *H^D+^*-*H^U-^* | 0.7147 | 0.2919 | 0.0349 | 0.0550 | 0.1758 | 0.4850 | 0.3156 | 0.0524 | 16.7862 |
| *H^S+^*-*H^U-^* | 0.8832 | 0.7954 | 1.3001 | 0.3258 | 0.1980 | 0.5213 | 0.6259 | 0.0227 | 6.4890 |
| *H^TD+^*-*H^U-^* | 0.0117 | 0.4139 | 0.3497 | 0.0457 | 0.0195 | 0.2996 | 0.3278 | 0.0162 | 3.0378 |
| *H^P+^*-*H^U-^* | 0.1793 | 1.4897 | 0.8592 | 0.3512 | 0.0959 | 3.1791 | 0.4790 | 0.0163 | 37.5338 |
| *H^U+^*-*H^T-^* | 2.5058 | 0.0619 | 0.0842 | 0.1260 | 0.2697 | 0.1405 | 0.1092 | 0.0033 | 6.5259 |
| *H^D+^*-*H^T-^* | 0.2501 | 0.3830 | 1.1589 | 0.4183 | 0.1328 | 0.1575 | 0.5111 | 0.0177 | 1.2500 |
| *H^S+^*-*H^T-^* | 0.1589 | 0.3127 | 2.0394 | 3.4538 | 0.1731 | 0.1562 | 0.3139 | 0.0974 | 2.9955 |
| *H^U+^*-*H^D-^* | 16.7169 | 0.2667 | 0.1195 | 0.3535 | 0.8385 | 0.3386 | 0.8702 | 0.0224 | 2.5019 |
| *H^T+^*-*H^D-^* | 6.0504 | 0.2318 | 0.0251 | 0.1445 | 2.0304 | 0.5913 | 0.0454 | 0.0484 | 8.5622 |
| *H^S+^*-*H^D-^* | 1.8838 | 1.0871 | 0.3240 | 0.3504 | 0.9579 | 0.1763 | 0.1143 | 0.0125 | 0.1861 |
| *H^U+^*-*H^S-^* | 0.1158 | 0.6606 | 1.0633 | 0.1905 | 0.4547 | 0.2819 | 0.1190 | 0.0463 | 0.3177 |
| *H^T+^*-*H^S-^* | 0.2212 | 0.5538 | 1.9237 | 0.5659 | 0.1295 | 0.2415 | 0.4224 | 0.0148 | 2.4971 |
| *H^D+^*-*H^S-^* | 0.2739 | 0.0852 | 2.9737 | 0.3020 | 0.0792 | 0.2049 | 3.2535 | 0.0222 | 11.1939 |
| *H^TD+^*-*H^S-^* | 1.2149 | 0.3225 | 0.1263 | 0.0330 | 0.1735 | 0.6112 | 2.2258 | 0.0235 | 6.3041 |
| *H^TD+^*-*H^SU-^* | 0.1546 | 0.4068 | 1.2635 | 0.6022 | 0.0959 | 0.1059 | 0.2071 | 0.0315 | 2.3067 |
| *H^T+^*-*H^SU-^* | 0.1156 | 0.1147 | 1.4138 | 0.0675 | 0.1403 | 0.1121 | 0.2842 | 0.0082 | 6.4429 |
| *H^D+^*-*H^SU-^* | 0.0166 | 0.0472 | 1.4221 | 0.0738 | 0.0475 | 0.0413 | 0.6829 | 0.0513 | 4.8801 |
| **Model Group II** (see also Methods; Table 1) | ***k*_1_*_a_***, *kaiA* transcription rate | ***k*_1_*_bc_***, *kaiBC* transcription rate | ***k*_2_*_a_***, *kaiA* translation rate | ***k*_2_*_bc_***, *kaiBC* translation rate | ***k*_3_*_a_***, *kaiA* degradation rate | ***k*_3_*_bc_***, *kaiBC* degradation rate | ***k*_4_*_a_***, KaiA degradation rate | ***k*_4_*_bc_***, KaiC degradation rate | ***λ***, Fold-change |
| *H^U+^*-*B^P-^* | 6.0784 | 0.0582 | 0.8307 | 5.0574 | 0.2016 | 0.0879 | 0.0953 | 0.0062 | 36.2894 |
| *H^T+^*-*B^U-^* | 1.5759 | 1.9117 | 0.0530 | 1.1691 | 0.0873 | 1.3904 | 0.0551 | 0.0146 | 1.2460 |
| *H^D+^*-*B^U-^* | 0.6173 | 0.1127 | 6.2108 | 0.5686 | 0.1252 | 0.3130 | 1.0928 | 0.0068 | 6.6167 |
| *H^S+^*-*B^U-^* | 8.3914 | 0.2790 | 0.1354 | 0.0600 | 5.2977 | 2.8590 | 1.4584 | 0.0366 | 79.3584 |
| *H^TD+^*-*B^U-^* | 0.0130 | 0.3929 | 1.8688 | 0.4250 | 0.0267 | 0.1761 | 0.0607 | 0.0072 | 0.2360 |
| *H^P+^*-*B^U-^* | 0.0527 | 0.0364 | 4.4306 | 0.6881 | 0.1079 | 0.3091 | 0.0484 | 0.0065 | 20.6309 |
| *H^U+^*-*B^T-^* | 0.5412 | 1.4694 | 1.4038 | 1.4189 | 0.4319 | 1.9894 | 0.1898 | 0.0696 | 1.4156 |
| *H^D+^*-*B^T-^* | 1.8481 | 0.1868 | 0.1094 | 0.1882 | 0.3544 | 0.8498 | 0.1010 | 0.0092 | 7.9984 |
| *H^S+^*-*B^T-^* | 0.6420 | 1.2120 | 0.7136 | 0.5927 | 0.4126 | 1.5835 | 0.3007 | 0.0538 | 1.6270 |
| *H^U+^*-*B^D-^* | 1.5322 | 0.0546 | 3.3557 | 1.1538 | 6.9459 | 0.2548 | 0.0685 | 0.0208 | 6.3954 |
| *H^T+^*-*B^D-^* | 0.4192 | 0.3124 | 2.3339 | 0.3446 | 2.1050 | 1.1289 | 0.2915 | 0.0318 | 7.1446 |
| *H^S+^*-*B^D-^* | 36.3139 | 0.3298 | 0.0822 | 9.8080 | 0.2563 | 0.2907 | 0.2764 | 0.1237 | 7.1220 |
| *H^U+^*-*B^S-^* | 0.0728 | 0.2416 | 1.0343 | 0.3110 | 0.0543 | 0.2769 | 0.2069 | 0.0114 | 2.2054 |
| *H^T+^*-*B^S-^* | 14.8031 | 0.9830 | 0.6655 | 0.2085 | 1.9525 | 2.0603 | 1.2143 | 0.0322 | 4.6549 |
| *H^D+^*-*B^S-^* | 4.5604 | 0.2408 | 1.3435 | 0.2155 | 0.8773 | 0.4977 | 0.9826 | 0.0172 | 5.8386 |
| *H^TD+^*-*B^S-^* | 1.0279 | 0.0743 | 21.1428 | 0.3430 | 0.2339 | 1.0948 | 0.7298 | 0.0694 | 0.0307 |
