# Supplementary material for: Revealing a Two-Loop Transcriptional Feedback Mechanism in the Cyanobacterial Circadian Clock
Source: PLoS Comput Biol. 2013 Mar 14;9(3):e1002966. doi: 10.1371/journal.pcbi.1002966 (PMC3597532; doi:10.1371/journal.pcbi.1002966)
Supplement: Text S1 — Supporting Information. More detailed information on choice of the activation and repression term in equation (5), cost function, binomial distribution calculation and simulations of kai mutants. (DOC) [file pcbi.1002966.s015.doc]

**Choice of activation and repression term in Eq. (5) in the main text**

Transcription of the *kaiBC* mRNA is based on simple activation and simple repression showcased in Table 1 from Bintu et al. [1]. We further assume that the transcriptional activator and the transcriptional repressor have opposite effects on *kaiBC* expression and they, therefore, do not compete for the same binding site upstream of the *kaiBC* promoter. Bintu et al. explain transcriptional control from a thermodynamic point of view such that interactions of activators, repressors, RNA polymerase and protein-protein interactions regulating gene expression are described by a single ‘regulation factor’ (F*_reg_*). Simple activation can thus be described through the mathematical form of expected fold-change of gene expression

.

The parameters are the effective *in vivo* dissociation constant (*K_A_*) between the activator and a promoter region and the enhancement factor (*λ*), which characterizes the degree of stimulation in transcription.

In this study, the simple activation involves a phospho-state of KaiC (U, T, D or S) from the hexamer pool, *C^H^* (see Methods). Case 2 in Table 1 [1] gives the mathematical form of the expected fold-change with

and *X* = *H^U^*, *H^T^*, *H^D^*, *H^S^*, *H^P^*.

For simplicity, *K_A_* was set to 1 resulting in

.

The mathematical form for simple repression is given by

with

$\left[ R \right]=Y$ and *Y* = *H^U^*, *H^T^*, *H^D^*, *H^S^*, *H^P^*, *B^U^*, *B^T^*, *B^D^*, *B^S^*, *B^P^*.

Here, the simple repression involves a phospho-state of KaiC (U, T, D or S) from the hexamer pool, $C^{H}$, and from the KaiBC pool, *C^B^* (see Methods).

The dissociation constant *K_R_* was set to 1 as well resulting in

.

Activators and repressors function at different times. Defining now that *k*_1_*_bc_* and *k*_3_*_bc_* are the transcription rate and degradation rate, respectively, transcription of *kaiBC* mRNA can be written as

,

which is identical to equation (5) of the manuscript.

**Optimization process**

In order to investigate whether a two-loop feedback model sufficiently explains the data we fitted each model to the measured data points derived from our own image analysis of Figure S2 from Murayama et al. [2], using a least-squares method. The values are given in Table S1. The cost function to be optimized is the sum of the least squares

(S1)

with

R = *kaiBC* mRNA,

U = unphosphorylated KaiC Protein/total KaiC, and

P = total phosphorylated KaiC protein/total KaiC.

**Binomial distribution**

The following binomial distribution is to show that using homogenous phospho-states of KaiC as responsible for the feedback regulation is a reasonable assumption.

We assume that every hexamer that contains threonine phosphorylated KaiC will contribute to the transcriptional feedback. (This is analogous for double phosphorylated, serine phosphorylated and unphosphorylated KaiC hexamers.) The probability for a hexamer T-KaiC and U-kaiC, D-KaiC or S-KaiC is a binomial distribution (special version of Eq. S16 in [3])

(S2)

We further assume that *i* T-phosphorylated KaiC lead to an *i*-fold binding affinity in comparison to a hexamer containing one T-phosphorylated KaiC. This expectation value of a binomial distribution reduces to

. (S3)

We therefore conclude that the binding affinity to the promoter is proportional to .

**Simulations of *kai* mutants**

In Figure 4 in the main text, overexpression of the *kaiA* gene induced at time of minimal *kaiBC* expression was simulated. For example, we have modeled an ox*kaiA* mutant by a 10-fold higher transcriptional rate

.

We have further introduced the heaviside function:

The KaiC translational rate *k*_2_*_bc_(t)* has been simulated analogously (Figures 5, S11).

**References**

1. Bintu L, Buchler NE, Garcia HG, Gerland U, Hwa T, et al. (2005) Transcriptional regulation by the numbers: models. Curr Opin Genet Dev 15: 116-124.

2. Murayama Y, Oyama T, Kondo T (2008) Regulation of circadian clock gene expression by phosphorylation states of KaiC in cyanobacteria. J Bacteriol 190: 1691-1698.

3. Brettschneider C, Rose RJ, Hertel S, Axmann IM, Heck AJ, et al. (2010) A sequestration feedback determines dynamics and temperature entrainment of the KaiABC circadian clock. Mol Syst Biol 6: 389.
